# Solution-Processed OLEDs: A Critical Review and Methodology Proposal for Stack Optimization

**DOI:** 10.3390/mi17020217

**Published:** 2026-02-05

**Authors:** Yassine Chiadmi, Paul-Vahe Cicek, Ricardo Izquierdo

**Affiliations:** 1 Department of Electrical Engineering, École de Technologie Supérieure (ÉTS), 1100 Notre-Dame St. W, Montreal, QC H3C 1K3, Canada; 2 Department of Computer Science, Université du Québec à Montréal (UQAM), 201 President-Kennedy Ave., Montreal, QC H2X 3Y7, Canada; cicek.paul-vahe@uqam.ca

**Keywords:** optoelectronics, OLED, solution-processed OLEDs, solvent orthogonality, Monte Carlo optimization, stack design methodology, Hansen solubility parameters (HSP), material compatibility, reproducibility

## Abstract

Solution-processed OLEDs represent a low-cost, scalable alternative to vacuum-deposited devices, particularly for flexible and large-scale applications. However, selecting compatible materials for each layer remains a complex task, further complicated by inconsistent documentation, solvent interactions, and limited reproducibility across the literature. This work presents a literature review and critical analysis of materials, solvents, and fabrication methods involved in solution-processed OLEDs, with particular attention to layer formulation, solvent orthogonality, and processing constraints. A Monte Carlo-based optimization framework is introduced as a proof of concept, aiming to formalize stack selection and explore viable combinations based on empirical constraints. The critical analysis highlights recurring issues in the field and advocates for a more structured, reproducibility-oriented approach to OLED design.

## 1. Introduction

Organic light-emitting diodes (OLEDs) are a type of electroluminescent device that offer several advantages over technologies such as light-emitting diodes (LEDs), liquid-crystal displays (LCDs) and quantum dot light-emitting diodes (QLEDs). Their self-emissive nature, potential for ultra-thin and flexible form factor, high contrast ratios, fast response times and wide viewing angles distinguish them from other display technologies. Among these, the most defining characteristic is their ability to directly emit light, eliminating the need for a backlight as used in LCDs. This allows for better control over contrast ratios and how deep blacks are.

OLEDs can be fabricated through two primary methods: vacuum evaporation and solution processing. This paper focuses on solution-processed OLEDs, detailing the fabrication steps, material selection criteria, and decision-making process behind different design approaches. Since the objective is both to provide a literature review and to introduce key concepts, attention is given to not only describing but also justifying each process and material choice.

In order to explain the choices made behind different designs, it is useful to establish current performance data points. While vacuum evaporation OLEDs currently dominate the commercial market with superior lifetimes and efficiency, solution-processed devices are in constant evolution. Optimized solution-processed fluorescent polymers have achieved luminous efficiencies of approximately 30 cd/A [1]. Solution-processed phosphorescent and thermally active delayed fluorescence (TADF) devices have helped surpass the 25% internal quantum efficiency limits of fluorescence. State-of-the-art solution-processed green phosphorescent OLEDs have demonstrated efficiencies exceeding 100 cd/A [2], while recent inkjet-printed multi-resonance TADF emitters have achieved EQEs over 13% with high color purity [3]. These values highlight that solution-processing is transitioning from a low-cost option to a promising high-performance alternative.

To achieve this, the core operating principles of OLEDs are first reviewed, followed by a discussion on performance metrics and characterization techniques. Next, fabrication techniques used in solution-processed OLEDs will be described and evaluated in terms of efficiency, precision and economic feasibility. Subsequently, an analysis of the functional layers and the materials most commonly used in each is presented. Materials are compared based on their electrical, optical, and mechanical properties, while also considering emerging alternatives. Finally, solvents are evaluated based on their orthogonality using their Hansen distance as a guideline, and thermal considerations are explored as well as their impact on the overall OLED performance.

Finally, a critical analysis of the literature will be presented, with a structured breakdown of the primary obstacles and proposed solutions. Comparative tables will be included throughout the paper to serve as quick reference tools, allowing for a direct comparison between materials, fabrication methods and performance metrics.

The goal of this work is to explore the current landscape of materials and processing methods for printed OLEDs, evaluate performance limits and critically assess reproducibility issues in the literature. To address these challenges, a Monte Carlo-based optimization method is proposed to formalize stack selection and support future design efforts. The analysis is meant to serve both as a proof of concept and as a foundation for more structured experimental development.

## 2. OLED Functionality

### 2.1. Introduction to OLEDs

OLEDs are electroluminescent devices that emit light when an electric current passes through organic semiconductor layers. Their pixels are self-emissive, so no separate backlight is required contrary to liquid-crystal displays. While inorganic LEDs are also self-emissive, LCD panels still maintain a significant market share in cost-sensitive television and monitor segments. However, OLED technology is rapidly expanding, particularly in applications requiring flexible substrates and unique form factors that rigid liquid-crystal displays cannot easily achieve, making them currently the thinnest practical backlight-free option. This enables a better control over image quality, especially in the contrast department, since backlight bleeding is eliminated. Common application include displays found on smartphones, TVs, VR headsets, lighting, and flexible electronics. The basic structure of OLEDs is composed of multiple layers, each serving a distinct role in charge transport and light emission. OLEDs can be classified based on their light emission direction: bottom emitting or top emitting. Bottom-emitting OLEDs emit light through the substrate and therefore require a transparent anode. Top-emitting OLEDs emit through the top electrode and require transparent or semitransparent cathodes. They can also be made more efficient thanks to a well-tuned microcavity effect, which refers to the constructive or destructive interference created by the light trapped between the OLEDs electrodes [4]. This section will explore the inner workings of OLEDs, detailing each layer and the behavior of electrons and holes through the device. The functionalities of OLEDs when compared to other electroluminescent devices are explored in Table 1.

### 2.2. Introduction to Solution-Processed OLEDs

Traditional OLEDs are fabricated using vacuum thermal evaporation. Organic materials are heated in a vacuum, which causes them to evaporate and condense onto a substrate, forming thin films. This process ensures high material purity, prevents unwanted reactions with air and allows for precise multilayer deposition, but requires specialized costly equipment [5].

In contrast, solution processing offers an alternative approach that takes advantage of the multilayer architecture of organic light-emitting diodes (OLEDs), enabling their fabrication in simplified device stacks compatible with top-emission configurations [9]. In this method, as shown in Table 2 each layer is prepared by dissolving organic semiconductors in suitable solvents and depositing the resulting solutions as thin films onto substrates using methods such as inkjet printing [8], spin coating [6], and screen printing [5]. A key challenge in solution processing is ensuring that successive layers do not dissolve or interfere with underlying layers, requiring careful formulation of inks to ensure solubility, stability and compatibility [10]. In an effort to address this challenge, recent studies have begun exploring machine learning algorithms to optimize ink formulations. This approach aims to significantly reduce experimental trial-and-error time [11].

Once deposited, films undergo drying and annealing to enhance film morphology and improve electronic properties [12].

**Table 2:** Comparison of fabrication methods for OLEDs. Summarized from [4,5,6,7,13,14,15].

Aspect	Thermal Evaporation	Solution Processing/Inkjet Printing
Fabrication Method	Multilayered architecture with top- and bottom-emission configurations	Multilayered architecture but simpler structures and are often fabricated in top-emission configuration
Layer Structure	Substrate, anode, hole-injection layer (HIL), hole-transport layer (HTL), emissive layer (EML), electron transport layer (ETL), cathode. May include charge blocking layers	Substrate, anode combined with HIL, HTL, EML, ETL, cathode. Layer structure is a main challenge.
Materials	Wide range of small molecule and polymeric materials.	Limited material selection due to solution processability, meaning that materials must be soluble, but layers must not affect each other.
Layer Deposition	Thermal evaporation and successive multilayer forming processes.	Inkjet printing, spin coating, blade coating, screen printing, roll-to-roll printing.
Layer Definition	Very well-defined layer stacks are achievable.	Difficulties in defining stacked layers due to potential dissolution, intermixing or penetration of underlying layers by subsequent layers. Necessitates solution orthogonality.
Film Morphology	Thin films produced by thermal evaporation are typically denser.	Thin films produced by a solution process are typically less dense, which can affect charge transport.
Performance	Typically exhibit higher luminous efficiency and longer lifetime.	Limited luminous efficiency, severe roll-off characteristics and shorter lifetime.
Encapsulation	Metal cans, glass covers sealed with epoxy adhesive, glass frit sealing.	Glass with thin-film encapsulation, organic/inorganic barriers. Stress engineering is very important to prevent cracking of flexible devices.
Cost	High fabrication cost due to vacuum equipment and material consumption.	Promising for low-cost and large-area production using printing technologies and reduced material consumption.

### 2.3. Structure of an OLED

**Figure 1:**
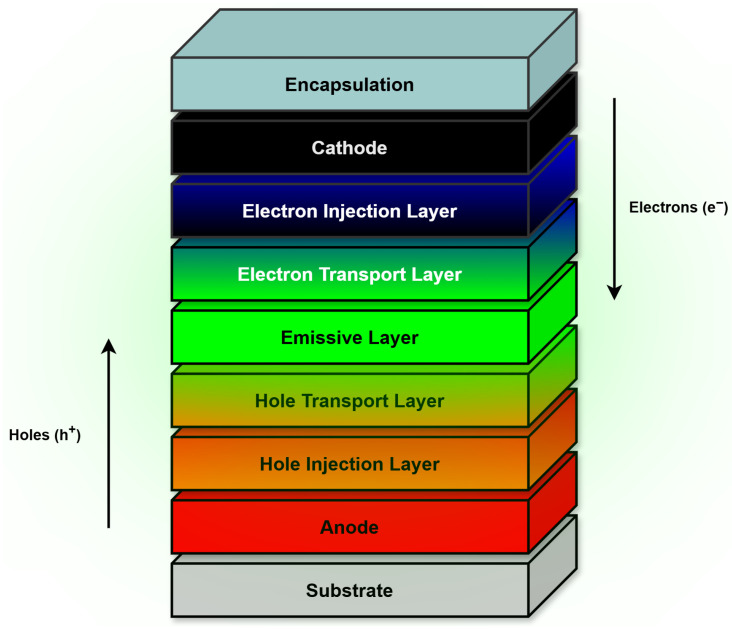
Solution-processed OLED layer stack.

Organic light-emitting diodes (OLEDs) consist of a multilayer stack built on a substrate that provides mechanical support, with each functional layer working together to convert electrical energy into light. A typical architecture, as illustrated in Figure 1, includes the anode, hole-injection layer, hole-transport layer, emissive layer, electron-transport layer, and cathode, where the hole and electron-transport layers play complementary roles in delivering charge carriers to the emissive layer.

The hole and electron transport layers play complementary roles by delivering holes and electrons, respectively, to the emissive layer. Consequently, the anode, HIL, and HTL must exhibit high conductivity with efficient hole transport but limited electron transport, whereas the ETL and cathode must be highly conductive while favoring electron transport and suppressing hole conduction. This charge-selective behavior improves device efficiency by ensuring that carriers are injected and transported in a controlled manner. When holes and electrons meet and recombine in the emissive layer, they form excitons that emit light. Optimizing material properties—such as energy-level alignment, charge-transport characteristics, and exciton management—enhances overall device performance by maximizing energy transfer and minimizing quenching losses.

In printed OLEDs, these layers are typically deposited using solution-based techniques such as inkjet printing and spin coating. The key layers include the following:

1. Anode: The anode serves as the electrode that injects holes into the organic layers. It must be transparent, highly conductive, and possess a high work function to enable efficient hole injection into the highest occupied molecular orbital (HOMO) of the organic materials. The anode is typically made of indium tin oxide (ITO) or, in some solution-processed architectures, PEDOT:PSS, which can act simultaneously as both the anode and the hole-injection layer (HIL) [6].
2. Hole-Transport Layer (HTL): The hole-transport layer facilitates hole transport from the anode or hole-injection layer to the emissive layer while preventing electron leakage [6]. It requires high hole mobility, low electron mobility, and good thermal and chemical stability. Its HOMO level should align closely with the anode’s in order to enhance compatibility and minimize charge injection barriers [7].
3. Emissive Layer (EML): The emissive layer is the primary light-emitting component, where electron–hole recombination generates photons through exciton decay. It is positioned between the hole-transport layer and the electron transport layer and must have good light emission properties, a low bandgap, and high photoluminescence quantum yield, which represents the ratio of emitted photons to absorbed photons in a material [6]. The choice of emissive layer materials directly affects emission color, contrast and efficiency, making material selection and deposition techniques critical in OLED performance [14].
4. Electron Transport Layer (ETL): The electron transport layer facilitates electron transport from the cathode to the emissive layer while blocking holes to improve charge balance, which refers to the equilibrium between injected electron and holes. It requires high electron mobility as well as good thermal stability and optical transparency. The ETL directly impacts OLED efficiency, charge balance and operating voltage [14].
5. Cathode: The cathode injects electrons into the organic layers and must have a low work function to minimize the electron injection barrier. It interacts with the electron transport layer to facilitate charge injection and requires proper energy alignment to maximize efficiency [7].

Printed OLEDs differ from conventional thermally evaporated OLEDs in fabrication techniques and material selection. Printing methods include inkjet, gravure, and screen printing, which enable scalable and cost-effective manufacturing [12,13].

As mentioned earlier, excitons are bound electron–hole pairs that can exist as either singlet or triplet states. Singlet excitons have antiparallel spins (S = 0), while triplet excitons have parallel spins (S = 1). Fluorescent OLEDs emit light exclusively from singlet excitons, limiting their internal quantum efficiency (IQE) to about 25% [9]. Phosphorescent OLEDs harness strong spin–orbit coupling to enable emission from triplet excitons as well, allowing a theoretical IQE of 100% [7], but requiring rare heavy metals such as iridium or platinum. Thermally activated delayed fluorescence (TADF) OLEDs also utilize both singlet and triplet excitons but achieve this by engineering a small singlet–triplet energy gap (ΔEST), which facilitates reverse intersystem crossing (RISC) to thermally upconvert triplet excitons into singlet states that can emit fluorescence [14]. Like phosphorescent OLEDs, TADF devices can theoretically reach 100% IQE, but they do so without relying on rare heavy metals, reducing material costs [16].

### 2.4. Charge Injection Mechanisms

Charge injection refers to the injection of electrons and holes from the electrodes through to the EML. In order to ensure a good charge propagation, it is important to properly choose materials that have work functions that align with adjacent layers. Since perfect alignment is not possible, charge injection layers, HIL and EIL, serve as ways to further improve energy level alignment and facilitate charge transfer [17].

Schottky emission is a mechanism where charge carriers are able to overcome and go over barriers by gaining enough thermal energy when an electric field is applied. Fowler–Nordheim tunneling is a quantum mechanical process where charge carriers go through a barrier when a sufficiently high electric field is applied. Under a high electrical field, the barrier becomes thinner and charges can go through it [18].

Those mechanisms are the main ways in which charges are injected at the electrodes and are not only governed by the strength of the electric field but by the physical properties of the materials themselves [18]. Both Schottky emission and Fowler–Nordheim tunneling become dominant when the local field approaches $5\cdot 10^{5}\;V\cdot {\mathrm{cm}}^{-1}$ or higher [18].

### 2.5. Charge Recombination and Exciton Decay

When electrons and holes meet in the EML, they form a quantum pair called excitons. Excitons are defined by their spin state. This refers to whether the electron–hole pair have antiparallel spins, which is a singlet exciton, or parallel spins, which is a triplet exciton. In the EML, in purely fluorescent systems, approximately 25% of excitons are formed in a singlet state, and 75% in a triplet state [5]. When excitons form, their lifetime is in the nanosecond range [5], then they decay. Radiative decay refers to electroluminescence, where light is produced, while non-radiative decay refers to non light-emitting processes and therefore reduce the efficiency of the OLED.

Although the statistical generation of singlet and triplet excitons follows a 1:3 ratio due to spin degeneracy, phosphorescent and TADF emitters allow for the radiative harvesting of the triplet population. This enables these devices to theoretically achieve 100% internal quantum efficiency, surpassing the 25% limit of conventional fluorescence [5].

In radiative decay, the exciton spin state will determine their decay process. Singlet excitons will decay through fluorescence, which is a short process in the nanosecond range [18]. Triplet excitons have a different decay process through phosphorescence, where they will go from a triplet state to a singlet ground state, which is the lowest energy state with paired spins. Phosphorescence is a longer process than fluorescence, in the micro to millisecond range, or even higher [14,18]. Thermally activated delayed fluorescence (TADF) is a mechanism where triplet excitons are first converted to singlet excitons, which then decay to the singlet ground state through fluorescence.

In non-radiative decay, several processes can occur. Quenching refers to when excitons lose their energy without emitting light. Defects, impurities and uneven emitter molecule distribution can lead to it. Exciton annihilation refers to instances where excitons interact with each other and convert their energy into heat or lesser energy photons. Recombination through trap states is also an issue in solution processing, where charges will be trapped within the layers or at their interfaces due to chemical degradation [4]. Excitons can also transfer their energy to adjacent layers, which are non-emissive, which leads to energy loss without light emission [8]. In order to prevent this issue, charge blocking layers can be used [17].

**Figure 2:**
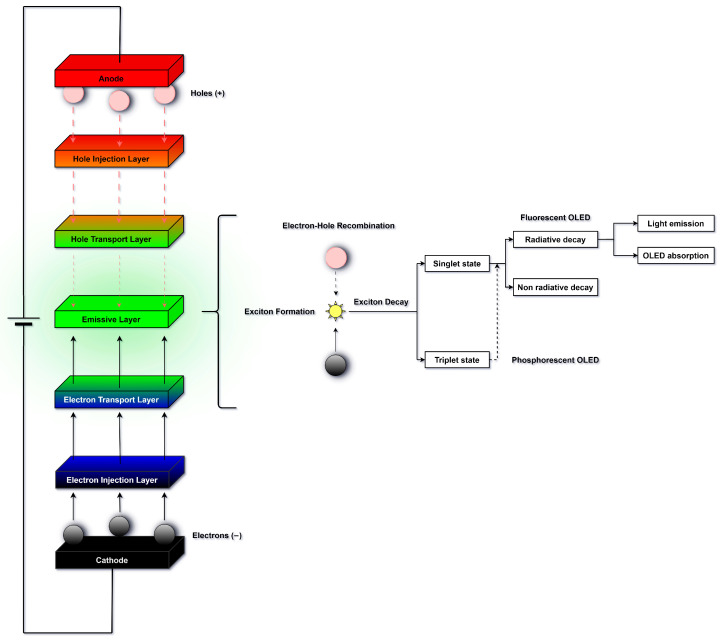
Electron–hole recombination diagram (adapted from Gaspar et al., [5]).

The overall mechanisms of exciton formation and decay are illustrated in Figure 2.

### 2.6. OLED Architectures and Configurations

OLED devices can be fabricated using several techniques, ranging from conventional vacuum thermal evaporation to printing-based methods comparable to paper printing, such as inkjet, roll-to-roll, or screen printing. OLED classifications generally depend on the materials and physical mechanisms responsible for light emission, as well as the device’s layer structure

1. Top Emission: Top-emitting OLEDs emit light through the top electrode rather than through the substrate. They therefore require a semitransparent cathode [17]. Thin Mg:Ag layers are frequently used because they can maintain adequate transparency at thicknesses of 10–15 nm. These layers are also commonly capped with ITO to improve conductivity and oxidation resistance while preserving transparency [5].
2. Bottom Emission: In bottom-emitting OLEDs, light is emitted through a transparent substrate—typically glass or a polymer [19]. The light still originates from the emissive layer [5] and must pass through the underlying thin-film structures, including thin-film transistors (TFTs) in active-matrix OLED configurations [4]. The anode is typically made of a transparent conducting oxide (TCO) [19], although thin semitransparent metal films may also be employed [5].
3. Passive Matrix: Passive matrix OLEDs (PMOLEDs) use a simple crossbar electrode layout where pixels are defined at the intersections [17]. They typically consist of an anode sheet, an organic layer and a cathode sheet. The color emitted will depend on the emissive layer formulation deposited at each row–column intersection [8], and the passive matrix addresses each row sequentially while applying column signals to select pixels [7]. This selects which predefined color pixel is turned on [20].
  PMOLEDs have a simpler structure than active matrix OLEDs (AMOLEDs) and are better suited for monochromatic displays [20]. They can be solution-processed using methods like spin coating and inkjet printing, which makes them promising for low-cost large-scale fabrication [21]. While they might be outperformed by AMOLEDs, they remain relevant due to their printable nature and lower complexity [20].
4. Active Matrix: Active matrix OLEDs (AMOLEDs) use TFTs to control each pixel individually, instead of selecting entire rows as in PMOLEDs. This enables higher contrast, resolution, faster refresh rates and better efficiency compared to passive matrix configurations [17]. Solution processing is being explored for the OLED stack and the organic TFT backplane. However, multilayer structures of this complexity introduce solvent compatibility and layer dissolution issues [10]. While solution-processed AMOLEDs are still lacking in performance when compared to vacuum evaporated AMOLEDs, they remain a promising avenue for large scale manufacturing.
5. Transparent, Flexible and Stretchable OLEDs
  - Transparent OLEDs: Transparent OLEDs emit light through both electrodes using fully transparent materials [17]. They require transparent materials or ultra-thin metals, silver nanowires or graphene as well as transparent encapsulation [6]. They are mainly used in smart windows, heads-up displays (HUDs) and see-through displays [17]. The main challenge is fabricating an efficient transparent cathode [6].
  - Flexible OLEDs: Flexible OLEDs are built on bendable substrates like PET, PEN or metal foils. Solution processing methods that are well suited to roll-to-roll fabrication offer a great potential for large scale fabrication since the substrate can be stored similarly to a roll of paper. Thin-film encapsulation is extremely important due to oxygen and moisture sensitivity [6]. Applications include wearables, rollable screens, curved displays and emerging technologies [5]. Mechanical stability and barrier performance are key issues.
  - Stretchable OLEDs: Stretchable OLEDs can deform without losing function [14]. They require intrinsically stretchable materials for all layers, including electrodes and emissive layer [14]. Solution processing allows patterning of flexible polymers [22] but fully stretchable solution-processed OLEDs are still in an experimental phase. Applications could include implantable devices or skin-conformable displays [14]. The main challenge is to maintain conductivity and emission under strain as well as resistance to repetitive mechanical stress [9].
6. Emerging OLEDs:
  - TADF OLEDs: TADF OLEDs are devices that use thermally activated delayed fluorescence to convert triplet excitons back to singlets without the use of heavy metals. This allows for very high efficiency, theoretically 100% IQE, using only organic materials [4]. Recent progress shows EQEs of up to 23.9% for blue emitters [17]. The main appeal is the higher performance with a lower material cost.
  - QD-OLEDs: QD-OLEDs use quantum dots as the emissive layer. The emission wavelength depends on QD size, which enables a high color purity and narrow spectra. They can be deposited by solution processing methods [8]. Quantum dots can also act as color converters when placed over white OLEDs. While EQEs are modest, QD-OLEDs offer a wide color gamut, tunability and potential for scalable manufacturing [17].
  - PeLEDs: Perovskite LEDs use perovskite crystals as the emissive layer. These materials have high photoluminescence yield, narrow emission, and tunable bandgaps. They are also fully solution processable and show a strong potential for color-tunable light sources. EQEs above 20% have been achieved in green, red and IR devices [14].

## 3. Performance and Characterization

This section introduces and defines the key performance metrics typically reported in the OLED literature.

### 3.1. Performance Metrics

1. Luminous Efficiency (cd/A): Luminous efficiency or Current Efficiency is defined as the ratio of luminance in candelas per square meter (cd/m^2^) multiplied by device area (*A*) to the current passing through the OLED. It reflects how effectively electrical current is converted to visible light [14]. Reported values for solution-processed OLEDs vary with color and deposition method, ranging from 1.4 to 23.8 cd/A at 100–1000 cd/m^2^ [23], with 5 cd/A for slot die coated devices [24]. However, direct comparison of these values requires caution, as reported efficiencies depend heavily on device architecture, layer thickness, and measurement conditions, which vary significantly between laboratory-scale reports and scalable or manufacturing protocols [6].
2. Power Efficiency (lm/W): Power efficiency measures light output (lm) per unit of electrical power (W). While solution processing enables low-cost routes to large scale fabrication [21], the main drawbacks lie in their efficiency due to layer intermixing [25], limited material options, and poor film morphology [26]. Power efficiency is directly affected by exciton quenching [14], charge imbalance [18], and interfacial incompatibilities. TADF and phosphorescent materials can help boost performance by taking advantage of triplet excitons [6].
3. Internal Quantum Efficiency (IQE): Internal quantum efficiency (IQE) measures how effectively injected carriers recombine to form photons in the emissive layer [14]. It depends on charge balance, exciton formation, and photoluminescence efficiency. Fluorescent emitters are limited to approximately 25% IQE, while TADF and phosphorescent materials help improve performance by harvesting triplet excitons, allowing theoretical IQEs of up to 100% [24]. In solution-processed OLEDs, IQE is also affected by layer intermixing, film morphology and dopant washing. Orthogonal solvents and crosslinking can help mitigate those issues [10].
4. External Quantum Efficiency (EQE): External quantum efficiency (EQE) measures how effectively injected carriers generate photons that exit the device [14]. It is defined as the product of the IQE and outcoupling efficiency, the latter referring to the fraction of generated photons that escape the OLED and contribute to visible light output [26]. Outcoupling is typically limited to 20–30% due to internal reflections [24]. EQE is affected by layer intermixing, film morphology, and material choices. Orthogonal solvents, interface engineering and optimized emitters help mitigate these issues.
5. Operational Lifetime (LT50/LT95): Operational lifetime refers to the time it takes for luminance to drop to a percentage of its initial value. LT50 (half-life) and LT95 (95% luminance retention) are common metrics. LT50 values are often reported at an initial luminance of around 1000 nits [4]. Lifetime is affected by film morphology, residual solvents, layer intermixing as well as charge balance and recombination efficiency [27]. The use of orthogonal solvents and crosslinking of layers helps improve lifetime.
6. Luminance (cd/m^2^): Luminance quantifies the brightness of an OLED in candela per square meter. It is typically evaluated using J–V–L curves, which record current density (J), applied voltage (V) and luminance (L), alongside current efficiency [24]. Higher luminance levels often accelerate device degradation. Film morphology, residual solvents, and layer intermixing can all affect luminance and its stability. Efficiency roll-off at high luminance is also a major challenge in solution-processed OLEDs.
7. Efficiency Roll-off: Efficiency roll-off refers to the decrease in luminous efficiency at high luminance levels. It is primarily caused by triplet-triplet and triplet–polaron annihilation, which are non-radiative processes occurring when triplet excitons interact with each other or with charge carriers, as well as by charge imbalance and dopant aggregation. These effects are exacerbated by poor film morphology and layer intermixing. Strategies to mitigate roll-off include improving charge balance, minimizing exciton quenching and optimizing dopant dispersion. Since high luminance is required in practical OLED applications such as displays and lighting, minimizing efficiency roll-off is a key factor in solution-processed OLED viability.
8. Turn-on Voltage ($V_{\mathrm{op}}$): Turn-on voltage is defined as the minimum voltage at which an OLED begins to emit light, typically measured at a luminance of 1 cd/m^2^ [6]. It should be as low as possible since it reflects the efficiency of charge injection into the device. A high turn-on voltage may result from injection barriers, poor film morphology or interfacial issues introduced during solution processing [5]. Proper alignment between electrode work functions and layer energy levels is also highly impactful. Doping of charge transport layers can enhance injection and reduce turn-on voltage. Film quality, residual solvents, and layer adhesion also influence turn-on voltage. OLED power consumption is directly influenced to the turn-on voltage [5].
9. Current Density: Current density measures the amount of current flowing through the OLED per unit area [5]. It is a key parameter for evaluating charge injection and transport. It is influenced by film morphology, residual solvents, and interfacial mixing [24]. Material choice directly affects conductivity and current flow. Poor interface definition can hinder charge transport, while the use of orthogonal solvents and crosslinking can improve it. Current density is also directly related to both luminance and current efficiency [24].
10. J–V–L Characteristics: J–V–L characteristics are used to describe the relationship between current density (J), applied voltage (V) and luminance (L) in an OLED [6]. They are used to evaluate charge injection, charge transport, and light emission. Efficiency metrics like candelas per ampere (cd/A) and lumens per watt (lm/W) are derived from these curves. Comparing J–V–L profiles helps identify performance limitations across different configurations [6].

### 3.2. Spectral and Colorimetric Properties

1. Color Quality (CIE Coordinates, CRI, CCT): Color quality is assessed using CIE (Commission Internationale de l’Éclairage) coordinates, the color rendering index (CRI) and the correlated color temperature (CCT). CIE coordinates define an OLED’s chromaticity, which describes the hue and saturation of the emitted light. It is a fundamental metric that quantifies human color perception. While it is extensively used to characterize specific emission colors of OLEDs [7], modern high-definition displays are often evaluated against the BT.2020 (Broadcasting Service Television 2020) standard [28], which defines a wide color gamut within the CIE 1931 standard [17] as illustrated by Figure 3. CRI measures how accurately colors are rendered, with values above 80 considered acceptable. CCT quantifies the warmth or coolness of white light [6]. Achieving precise color performance remains challenging due to solvent intermixing and limited orthogonality.

**Figure 3:**
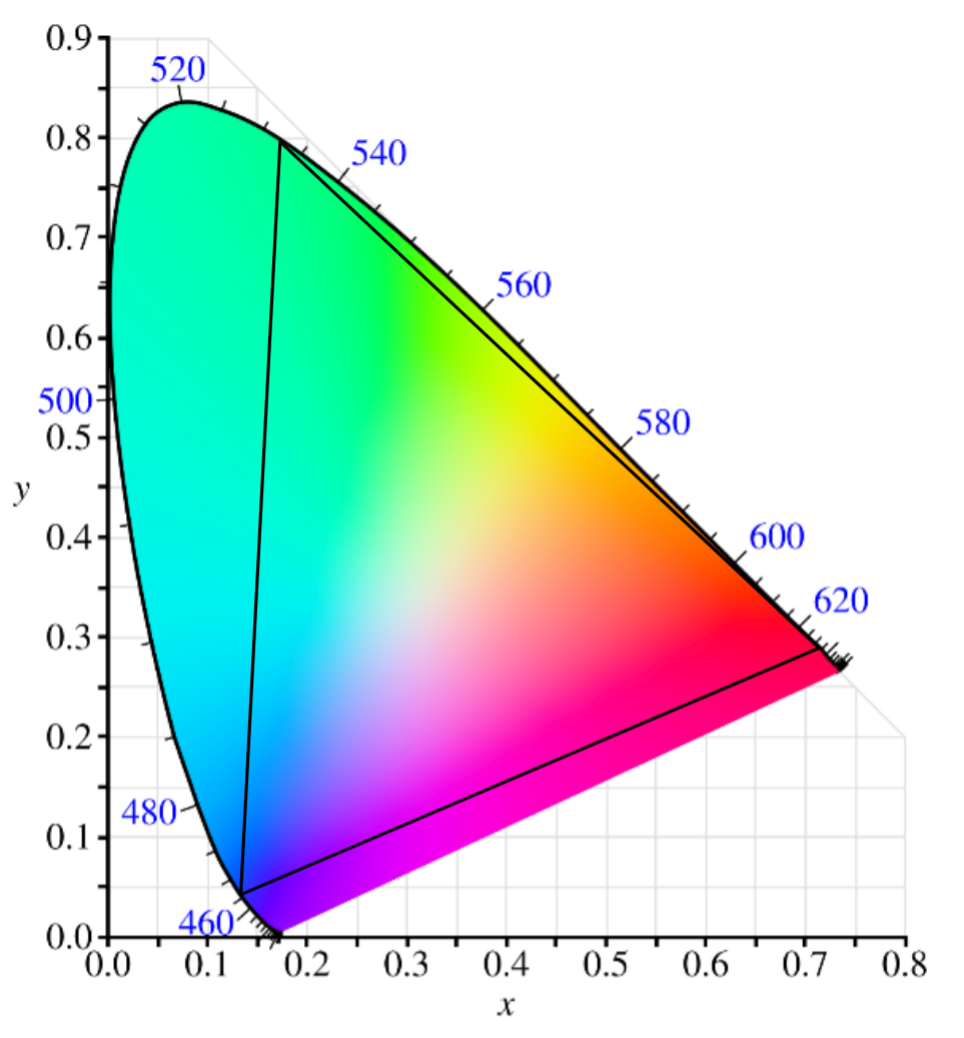
CIE 1931 chromaticity diagram illustrating the BT.2020 color gamut. Adapted from public domain image “CIE1931xy blank” by BenRG [29]. The colored area represents the visible spectrum, and the blue numbers along the curve indicate the wavelengths in nanometers (nm).
2. Electroluminescence (EL) Spectrum: The electroluminescence (EL) spectrum shows the emitted light intensity as a function of wavelength. The main peak wavelength ($\lambda_{EL}$) corresponds to the emission of the active layer and reflects the HOMO-LUMO energy gap, which is the energy difference between the highest and lowest occupied molecular orbitals of the emitter. Peak intensity increases with voltage beyond the turn-on threshold [6]. The spectral width reflects color purity, with narrower spectra indicating better color definition. Measuring differences between the EL and photoluminescence (PL) spectra helps confirm that emission originates from the EML and not other layers [30]. Processing conditions and solvent choice can affect the spectrum. However, optimized solution processing methods have been shown to match spin-coated results [30]. The EL spectrum is also tied to chromaticity and color temperature and remains one of the key tools to evaluate and tune the optical performance of OLEDs.

### 3.3. Morphology and Processability

Film morphology and uniformity are critical aspects of solution-processed OLEDs, directly affecting efficiency, emission quality, and stability [31]. Non-uniform films lead to current leakage, uneven luminance and short lifetimes. Morphology is influenced by ink formulation, solvent choice, drying dynamics, and post-treatments like annealing. Solvent intermixing can cause layer disruption and poor interface quality [32]. The use of orthogonal solvents and crosslinking layers helps improve stack definition. Coffee-ring effects [12] and crystallization are also common issues in inkjet printed films. Tools like ellipsometry and atomic force microscopy (AFM) are used to assess film roughness, thickness, and interfacial quality [33].

## 4. Solution Processing Techniques for OLEDs

Solution processing techniques encompass a wide range of deposition methods for creating thin films. These methods rely on the formulation of inks or solutions that contain the desired active materials, typically dissolved or dispersed in suitable solvents. Each technique offers specific advantages and limitations in terms of resolution, layer uniformity, scalability, and compatibility with multilayer architectures. This section presents the most commonly used techniques for solution-processed OLED fabrication, along with a few emerging approaches.

### 4.1. Common Techniques

1. Inkjet Printing: Inkjet printing is a fast, high-resolution deposition method that allows minimal material waste by only using the required amount of ink. It is non-contact and mask-free and is therefore useful for multilayer structures [12]. Inkjet printing requires high accuracy from the printer, but drop-on-demand piezoelectric heads enable precise control of droplet size and placement. Ink jettability is critical, as it determines how reliably droplets can be extruded from the printer head [30]. Ink viscosity is another key parameter. A maximum value of approximately 20 mPa · s is recommended to limit nozzle blockage. In addition, solvents with a higher boiling point can be used to reduce coffee-ring effects [8]. Orthogonal solvent systems and crosslinking strategies are often used to preserve layer separation. Inkjet printing has been used to fabricate solution-processed OLED devices in ambient conditions [23] and is compatible with roll-to-roll processing. While conventional inkjet printing is still limited by droplet size resolution, Electrohydrodynamic jet printing has recently emerged as a manufacturing technique that uses electric fields to pull ink from the nozzle rather than pushing it. This enables sub-20 μm resolution and work with higher viscosity inks [34].
2. Spin Coating: Spin-coating is a commonly used technique for depositing thin films of organic materials from solution, allowing for quick testing of different formulations [35]. The process consists of depositing a liquid onto a flat surface and spinning it at a high speed to spread the material into a uniform film. The film thickness depends on temperature, spin time, spin speed, viscosity, and solvent volatility [17]. While effective for lab-scale optimization, spin-coating wastes material and is less suitable for large area or multilayer OLED fabrication as other methods.
3. Slot Die Coating: Slot die coating is a pre-metered deposition technique, meaning the ink volume is precisely controlled before deposition. It is compatible with roll-to-roll processing and suited for large area thin films [24]. Film thickness is controlled by ink flow rate, coating speed and the width of the slot die head [35]. Slot die coating offers high ink utilization, low contamination risk, and can accommodate a wide range of ink viscosities. However, it suffers from non-uniform deposition and layer intermixing. Therefore, optimizing formulation parameters is essential, and the use of orthogonal solvents and crosslinkable materials helps preserve stack integrity. This method is considered highly promising for the upscaling of OLED production [31].
4. Blade Coating: Blade coating is a technique in which a blade is used to spread a liquid film across a substrate, enabling the formation of uniform layers over large areas [24]. The film thickness, ranging from nanometers to micrometers, can be controlled by adjusting the blade speed, gap height and ink properties [17]. Although less scalable than other methods, blade coating is commonly used in research environments and has been shown to work in a semi-continuous laboratory system on a small scale [32]. It has been used in multilayer white OLED fabrication [5]. While less prominent than inkjet or slot die methods, it remains a simple and versatile technique.
5. Roll-to-Roll Processing (R2R): Roll-to-roll is a continuous manufacturing method widely used for the production of flexible electronics [24]. It transports a flexible substrate through sequential stations that perform printing, coating, drying and curing steps [31]. Key aspects include substrate speed, drying temperature and coating precision, all of which must be optimized to ensure film quality. In multilayer stacks, process parameters must be finely tuned to prevent interlayer mixing and preserve layer integrity [24]. Roll-to-roll enables the integration of various deposition techniques into a unified, high-throughput manufacturing system suitable for industrial-scale production [24].
6. Gravure Printing: Gravure printing is a high-throughput solution deposition method compatible with roll-to-roll production [9]. It uses an engraved cylinder to transfer ink onto the substrate via capillary, with film thickness and pattern resolution controlled by the cell dimensions and ink properties [6]. It enables large-area coating [35] and is compatible with flexible substrates. Its advantages include scalability and high processing speed [5], though challenges remain regarding ink formulation, film uniformity, and device emission efficiency. It is a strong candidate for industrial solution-processed OLED fabrication [35].
7. Flexographic Printing: Flexographic printing uses a flexible relief plate, along with an engraved cylinder known as an anilox roll, to transfer ink onto a substrate [5]. It supports a wide range of ink types and offers better resolution, sharper edge definition and more consistent lines than gravure printing, although it remains less precise than inkjet printing [6]. It is also compatible with roll-to-roll production. While flexography is less suited for high-precision multilayer fabrication, it remains viable for large-area OLED applications where a very high resolution is not critical [5].

### 4.2. Emerging Techniques

1. 3D Printing: 3D printing is a solution processing method for soft electronics that enables layer-by-layer additive deposition. It uses conductive or semiconductive inks to deposit layers on non-planar surfaces, potentially enabling more complex geometries than those achievable with conventional printing methods [9]. Proposed applications include wearable electronics, robotics and bioelectronics. Challenges include lower carrier mobility, poor layer uniformity, and resolution limits, with solvent optimization and nozzle design being explored as solutions. While promising, 3D printing remains at an experimental stage and requires further refinement before it can be considered for larger scale OLED fabrication [9].
2. Electrodeposition: Electrodeposition is a method where chemical processes involving electron transfer, known as redox reactions, deposit thin films on electrode surfaces [20]. The process offers precise control through applied voltage or current and is used to form conductive or semiconductive organic layers. A key method is electropolymerization, where stable material clusters are grown into continuous films ranging from linear (1D) chains to more complex 3D networks. Electrodeposited films can be luminescent [20]. In contrast to most solution-based techniques, film formation is electrochemically driven, rather than relying on solvent evaporation. While electrodeposition has shown promise for OLED fabrication, challenges remain regarding interface control and overall stack integration [20].

## 5. Materials and Processes for OLEDS

**Figure 4:**
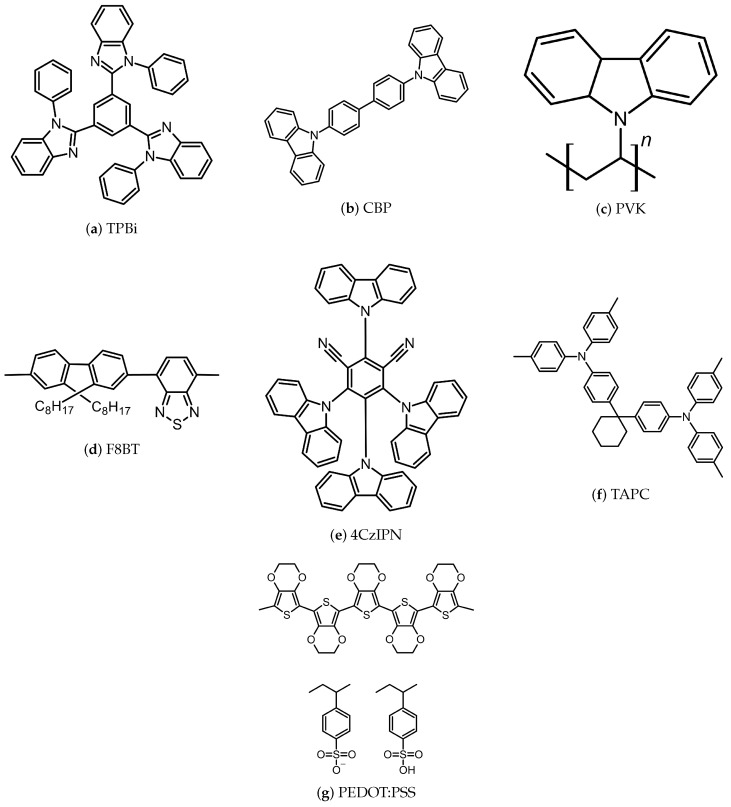
Chemical structures of representative materials discussed in Section 5.3, Section 5.4, Section 5.5 and Section 5.6.

Materials used in printed and solution-processed OLEDs span a broad spectrum of organic and inorganic compounds, with polymers and metals being the most prevalent representatives of each class. While metals generally offer more limited tunability and application scope, polymers can be chemically modified, functionalized, or blended—enabling tailored electrical, optical, and mechanical properties. Consequently, many modern OLED formulations rely on hybrid materials, copolymers, and controlled doping strategies to optimize performance, efficiency, and stability. This section reviews the most commonly used materials for each solution-processed OLED layer, prioritizing undoped or lightly doped formulations unless otherwise noted. When relevant, materials that can serve multiple roles across different layers are also highlighted. To assist in visualizing, the chemical structures of some representative materials discussed in Section 5.3, Section 5.4, Section 5.5, Section 5.6, Section 5.7 and Section 5.8 are provided in Figure 4.

### 5.1. Encapsulation Process and Materials

- Atomic Layer Deposition (ALD): Atomic layer deposition is an encapsulation method used for printed and solution-processed OLEDs to protect them from moisture and oxygen degradation [5]. It is used to deposit uniform inorganic films like SiO_2_, Al_2_O_3_, ZrO_2_ and TiO_2_ at low temperatures, which ensures a coverage without damaging the underlying organic layers [15].
- Solution-processed polymers: Solution-processed polymers are used as emissive, charge transport, interlayer and encapsulation materials due to their scalability, cost-effectiveness and tunable electrical properties. They are, however, prone to phase segregation which leads to uneven electrical and optical properties, and to solvent compatibility issues [6]. Commonly used light-emitting polymers such as PPV and MEH-PPV integrate charge transport and emissive functionalities but have limited efficiency. Materials such as PEDOT and PANI offer conductivity and mechanical stability and require additional processing to prevent the dissolution of underlying films. Solution-processed polymers are primarily deposited using spin coating and inkjet printing [5].
- Hybrid Organic/Inorganic: Hybrid organic/inorganic materials combine the barrier properties of inorganic oxides with the flexibility of polymers [15]. Inorganic layers such as SiO_2_, Al_2_O_3_, ZrO_2_ and TiO_2_ are deposited via ALD, while organic layers, including CYTOP, polymerized hexane and alucone, help improve adhesion and mechanical stability. These multilayer structures enhance barrier performance and protect the OLEDs against moisture and gases, while optimizing mechanical reliability. Emerging materials such as bacterial nanocellulose are also being explored as biodegradable, flexible materials that could improve both stability and sustainability while maintaining high flexibility [36].

### 5.2. Substrates

- Bacterial Nanocellulose (BNC): A polymer synthesized by microorganisms such as *Komagataeibacter*. These bacteria are cultivated in a carbon-, nitrogen- and mineral-rich environment where they produce an interwoven network of nanofibers. As a result, BNC exhibits insolubility, rapid biodegradability, good tensile strength, elasticity, durability while being non-toxic and non-allergenic. Its optical transmittance is inherently low but can be enhanced by filling structural voids combining it with other materials. Its flexibility makes it suitable as a biomaterial for a flexible substrate and is regarded as a viable alternative for glass in rigid OLEDs [36].
- Polyethylene Terephthalate (PET) and Polyethylene Naphthalate (PEN): Polyethylene terephthalate (PET) and polyethylene naphthalate (PEN) are widely used as flexible substrates due to their high optical transparency, mechanical strength, and solvent resistance [5]. Both support efficient light output but require low deposition temperature techniques due to their limited thermal stability. Their weak barrier properties against water vapor and oxygen necessitate surface modifications to enhance surface wettability and processing compatibility. Compared to PET, PEN offers slightly better thermal stability and mechanical durability, making it more suitable for higher temperature processes [6].
- Glass: Glass is a widely used substrate due to its stability, high glass transition temperature, and excellent optical and chemical properties [6]. It offers high clarity, low roughness and thermal stability while being resistant to various solvents. However, its rigidity limits its applicability in flexible devices [5,36].

### 5.3. Electron Transport Materials

- PFN-Br: A bromide salt derivative of PFN, PFN-Br (Poly(9,9-bis(3′-(N,N-dimethyl)-N-ethylammonium-propyl-2,7-fluorene)-alt-2,7-(9,9-dioctylfluorene))dibromide) is a polymer soluble in water, DMF, DMSO and alcohol that has the ability to form uniform thin films [37]. PFN-Br has been used as an ETL material in a fully solution-processed OLED structure [6].
- TPBi (1,3,5-tris(N-phenylbenzimidazol-2-yl)benzene): TPBi is an organic molecule soluble in alcohol, enabling the formation of uniform thin films. It can act as both the ETL and an exciton blocking layer (EBL), reducing non-radiative decay and in turn improving OLED efficiency. However, its use in blue phosphorescent OLEDs is limited due to triplet exciton quenching [4].
- PO-T2T: PO-T2T is a small molecule material soluble in alcohol, which helps enable good thin-film formation. It is primarily used as an ETL material. Devices using PO-T2T as the ETL have shown good performance close to vacuum-deposited counterparts, with differences in operating voltage and some leakage current [25].
- Zinc Oxide (ZnO): ZnO is a solution-processable inorganic material that is mainly used as an ETL due to its high electron mobility and low work function. It has been used in high-efficiency QLEDs as well as solution-processed OLEDs. However, its electron mobility is one to three orders of magnitude larger than that of common organic HTL materials like PVK, Poly-TPD, and TFB, which can cause excess electron accumulation and lead to charge imbalances [8].
- Titanium Dioxide (TiO_2_): Titanium dioxide is an inorganic solution processable material that is investigated as an EIL and ETL material. Its high work function facilitates electron injection. Research involving porous TiO_2_ nanocrystals has also demonstrated potential as ETL materials [7].
- PANI (Polyaniline): Polyaniline is a conductive polymer used for its electrical properties and processability. It can function as a hole or electron transport layer (HTL/ETL), facilitating charge transport. PANI is often combined with other materials such as polymeric nanohybrids or nanocomposites to enhance OLED performance. Its flexibility and solubility make it suitable for integration into flexible OLEDs as well as ink formulations for printed electronics [7].

### 5.4. Cathode Materials

- Silver Nanoparticles (Ag-NPs): Silver nanoparticles (Ag-NPs) are used in printed and solution-processed OLEDs to enhance light out-coupling efficiency and serve as electrode materials [6] while also improving light extraction through plasmonic effects and light scattering [26]. Silver nanoparticles can be incorporated into cathodes or hybrid electrodes, such as silver nanowires combined with graphene, for flexible OLEDs. They can be deposited via inkjet printing or thermal-assisted self-aggregation [5,19]. Their size and formulation affect sintering behavior and film properties, which define the possible low-temperature windows for solution processing [38].
- Magnesium–Silver alloy (Mg-Ag): Mg-Ag alloy is used in printed and solution-processed OLEDs due to its low work function, enabling efficient electron injection into the emissive layer [6]. The alloy offers greater stability while maintaining good electrical performance when compared to pure metals. It is commonly used in flexible OLEDs and transparent OLEDs, where it contributes to the device’s flexibility [13,36].

### 5.5. Emissive Layer (EML) Materials

- TADF (thermally activated delayed fluorescence) Materials: TADF materials are used in OLEDs in order to achieve high light emission efficiency without using heavy metals. They enable nearly 100 % Internal Quantum Efficiency (IQE) by converting the non-radiative triplet excitons into emissive singlets. Common TADF materials include 4CzIPN and ACRXTN, often paired with CBP to improve performance. These materials can also be formulated as inks for inkjet printing [23].
- Small Molecule Solution Materials: Small molecule solution materials are low molecular weight compounds that dissolve in various solvents, making them suitable for ink formulation and inkjet printing. Used as EML materials in OLEDs, they offer high carrier mobility, easier synthesis, purification and improved reproducibility compared to polymers. Solution processing of materials such as 2PE-PPF and DPE-PPF can provide cost and scalability advantages over thermal evaporation. DuPont has developed solution processing techniques enabling the integration of water-based hole-injection layers (HIL) on small molecule emitters for high-resolution smooth film deposition [13].
- F8BT: F8BT is a polyfluorene copolymer used as a green emitting light-emitting polymer (LEP) in polymer-based OLEDs. It exhibits high electron mobility but tends to accumulate excitons because of unipolar hole transportation [19]. TPD (*N*,*N*′-di(3-methylphenyl)-*N*,*N*′-diphenyl-(1,1′-biphenyl)-4,4′-diamine) can be added to mitigate this effect, which will improve charge distribution. In addition, F8BT is soluble in xylene, which allows it to be spin-coated onto inorganic layers like TiO_2_ and ZnO while being resistant to subsequent solution-processed layers [20].
- Polyvinylcarbazole (PVK): PVK is a polymer used as a hole-transport layer (HTL), emitting layer (EML) or host material for phosphorescent dopants. It can be used to modify polymeric hole-injection layers (HILs) to reduce exciton quenching and therefore serve as an energy barrier bridge layer [4]. PVK-based materials can also be crosslinked for improved stability and exhibit good surface uniformity with resistance to common organic solvents [25].
- MEH-PPV: MEH-PPV is used as an orange-red light-emitting polymer in PLEDs. It can be solution processed and spin coated onto polyaniline anodes without damaging underlying layers, which enables multilayer fabrication [4]. However, its performance is limited by low hole mobility and a large bandgap, which limits its efficiency in PLED applications [19].
- Quantum Dots (QDs): Quantum dots are semiconductor nanocrystals that can be tuned to modify their emission properties, which makes them a promising candidate for electroluminescent displays. They consist of a core, a wide-bandgap shell and organic ligands [19]. They are used for manufacturing QLEDs, which share structural similarities with PLEDs, allowing for compatible fabrication methods. Inkjet printing enables precise deposition of QD layers [8].

### 5.6. Host Materials

- CBP: CBP (4,4′-bis(N-carbazolyl)-1,1′-biphenyl) is commonly used in solution-processed OLEDs. As a host material for emissive materials, it influences charge balance within the emissive layer (EML) and affects both device efficiency and lifetime. Due to unequal electron and hole mobility, it is often combined with materials like TCTA in mixed-host systems, which reduces the hole-injectionbarrier and enhances luminance and efficiency. Additionally, CBP helps mitigate the efficiency roll-off at high luminance and provides resistance to organic solvents, which makes it a strong candidate to ensure stability during the solution-coating of charge transport layers [4].
- PMMA: PMMA (Polymethyl methacrylate) is widely used in printed and solution-processed OLEDs due to its optical and electrochemical properties, flexibility and mechanical strength. It is highly transparent and provides good weather and scratch resistance. It can serve as a host matrix for emissive materials, where it is often combined with small molecules or polymers. It is also used as a matrix for nanocomposites, where metal oxides enhance its properties. Notably, reinforcement with lead oxide (PbO), has been shown to further improve its transparency [19].

### 5.7. Hole Transport Materials (HTM)

- PEDOT:PSS: In addition to its use as a combined anode and HIL materials, many designs simplify the layers by combining the anode, HIL and sometimes the HTL using PEDOT:PSS. This considerably reduces the solvent orthogonality issues between layers, as many other HTL materials use the same solvents as the most common EML compounds.
- Polyaniline (PANI): Polyaniline is a conductive polymer with tunable electrical properties and good solubility. As an HTL, it is compatible with flexible OLED architectures and printable formulations [7]. It is often combined with other materials such as polymeric nanohybrids or nanocomposites to enhance performance.
- TPD: TPD is a hole transport material commonly used in OLEDs due to its high hole mobility. It facilitates charge transport from the anode to the emissive layer, improving charge injection and device efficiency. However, its low glass transition temperature ($T_{g}$) makes it unsuitable for solution processing, as it crystallizes easily in thin films. TPD can be used as a hole trap when added to emissive layers like F8BT, improving charge balance and reducing exciton accumulation [6].
- TAPC: TAPC (1,1-bis[(di-4-tolylamino)phenyl]cyclohexane) is a hole transport material commonly used in printed and solution-processed OLEDs. It facilitates hole transport and can form interfacial exciplexes with materials such as BTPS. This interaction improves charge transfer and reduces exciton quenching. TAPC is also used in solution processing, where it can enhance film properties when blended with PVK [23].

### 5.8. Anode Materials

- PEDOT:PSS: PEDOT:PSS, or poly(3,4-ethylenedioxythiophene) polystyrene sulfonate, is a conductive water-soluble polymer [12]. It is widely used as both a hole-injectionlayer and transparent anode in the production of OLEDs due to its high electrical conductivity and thermal stability. Its properties also make it a strong alternative to indium tin oxide (ITO) in printed electronics. Beyond its inherent transparency and high conductivity, PEDOT:PSS can be tuned to optimize its electrical, optical, and structural properties, broadening its range of applications. Its water solubility enables its formulation as an ink, making it particularly advantageous in inkjet printing, enabling uniform thin-film deposition [22].
- Indium Tin Oxide (ITO): Indium tin oxide is a widely used anode material due to its high work function, which facilitates hole injection into the OLED stack. It offers excellent conductivity, high transparency and strong surface adherence, making it suitable for deposition onto both glass and flexible polymer substrates [6]. It is less flexible than PEDOT:PSS, and alternatives are often explored due to its mechanical limitations [7,39].

### 5.9. Performance Overview

To analyze and benchmark the current capabilities of solution-processed OLEDs, it is essential to review the performances achieved in recent literature. Table 3, Table 4 and Table 5 summarize the performance of representative devices across the main emitter configurations: fluorescence, phosphorescence and TADF. These entries highlight the device architecture, deposition method, maximum luminous efficiency and maximum EQE. While device stability is a critical parameter, it is omitted from the following tables due to the lack of standardized reporting in the literature.

### 5.10. Doping

Doping increases charge carrier density in transport layers, improving injection and reducing voltage losses. Dopants are mixed into the ink before deposition. In transport layers, doping targets conductivity, while in the emissive layer, it aims to enhance emission efficiency. Solvent compatibility and dopant stability are the main challenges, with n-dopants, which donate electrons to the host material, being especially difficult to stabilize. Dopant washing is also a risk when dealing with multilayer structures. This refers to unintentional removal or redistribution of dopant molecules when depositing a layer on an underlying one. Strategies to mitigate this include the use of orthogonal solvents, crosslinking or using dendrimers, which are large molecules used to improve solvent resistance [5].

### 5.11. Common Metrics

- Work Function: The work function defines the energy needed to remove an electron from a material’s surface and directly impacts charge injection [18,51]. Efficient injection therefore requires alignment between electrode work functions and the HOMO/LUMO levels of the layers [5]. Anodes require high work functions, while cathodes require low ones [18]. HOMO (highest occupied molecular orbital) refers to the highest energy orbital containing electrons that can be excited to a higher energy level, while the LUMO (lowest unoccupied molecular orbital) is the lowest energy orbital that can accept an electron. Their energy difference determines the photon energy, or color, that the emitter can produce. Misalignment increases operating voltage and in turn reduces efficiency. Solvent interactions and layer dissolution also have a direct impact on work functions. It is therefore a critical aspect of OLED design to optimize work function alignment while accounting for potential issues due to solution processing.
- Conductivity: Conductivity enables charge injection and transport throughout the OLED stack and directly affects voltage and efficiency. Electrodes like ITO and PEDOT:PSS [12] offer high conductivity, with some materials being tunable with additives [52]. Conductivity is affected by solvent choice, film morphology, interlayer mixing and material purity [5]. Doping can be used to increase carrier density, which improves injection and lowers voltage. Orthogonal solvents and crosslinking help preserve conductivity.

Table 6 summarizes the key electronic properties of some materials discussed in this section. It lists the reported work function and conductivity values, which are critical parameters for ensuring proper energy level alignment and efficient charge transport in solution-processed OLED stacks. Note that where direct work function measurements are unavailable, HOMO values are provided as an approximation to aid in the estimation of injection barriers.

### 5.12. Gaps and Limitations in the Existing Literature

Although the literature presents a wide variety of materials for printed and solution-processed OLEDs, a persistent limitation is the lack of comprehensive justification for the full device stack. Most studies highlight a single novel layer or material yet rarely explain how this addition influences the adjacent layers or the overall device architecture. As a result, readers must implicitly assume that issues of compatibility, reproducibility, and practical feasibility have been addressed.

In many cases, the rationale for selecting neighboring layers—or the way a newly introduced layer interacts with the complete OLED stack—is only superficially discussed. Device lifetimes are usually reported, but the deeper effects of material combinations, interfacial phenomena, and interlayer interactions are seldom analyzed in detail.

Furthermore, while some works briefly touch on scalability or the transition to large-area manufacturing, comprehensive evaluations of processing tolerances, manufacturing constraints, or integration challenges remain limited, particularly in materials-centric research. Many studies focus on demonstrating a novel concept without assessing its readiness for real-world production.

Comparative analyses also tend to be narrow in scope. They typically emphasize performance outcomes without addressing rejected alternatives, design trade-offs, or the reasoning behind specific material choices. This omission leaves readers without critical context and limits the reproducibility of reported results. As noted by Merklein et al.,

“to the best of our knowledge, there are no previous comparative works where a qualitative and quantitative comparison of the application of different industrial solution processes and techniques for the fabrication of OLEDs, which also includes device performance investigation, are reported” [35].

These limitations do not reflect a lack of expertise within the field but rather a gap in reporting practices especially in research-oriented publications. Improved transparency regarding material selection, stack design, and decision-making processes would enhance reproducibility and support a more systematic advancement of OLED technologies.

## 6. Solvents and Solvent Orthogonality in Solution-Processed OLEDs

Solvent selection is a critical aspect of designing solution-processed OLEDs. Beyond simply dissolving the target materials, each solvent must be chosen with the full multilayer device architecture in mind. Because OLEDs are built through sequential deposition of solution-processed films, ensuring that each new layer can be deposited without damaging the ones beneath it is essential.

The central challenge is solvent orthogonality: solvents used for upper layers must not dissolve, swell, or otherwise disturb previously deposited layers. Any partial or complete dissolution leads to intermixing, rough interfaces, and degraded device performance. Achieving orthogonality therefore requires solvents that dissolve the desired material efficiently while remaining chemically and physically non-interactive with underlying films.

In this section, commonly used solvents are presented through an orthogonality matrix to highlight the most compatible combinations. Hansen solubility parameters (HSP) are employed to quantify solvent–material interactions and facilitate rapid identification of orthogonal solvent pairs. This framework provides a systematic approach for determining which solvents enable reliable sequential deposition.

Additionally, a representative solution-processed OLED stack is analyzed to demonstrate practical solvent-selection strategies, with HSP values used to evaluate layer-to-layer compatibility and guide the formulation of robust, orthogonal solvent systems.

Of note is the use of metal nanoparticles, which are dispersions rather than solutions. Dispersions differ from solutions in that the material is not dissolved but suspended within a carrier fluid.

### 6.1. Crosslinking

Crosslinking is used to make layers solvent-resistant and prevent intermixing during sequential deposition. It involves making materials react with crosslinking agents that form chemical bonds after a thermal or photochemical treatment. This enhances interfacial definition and improves device stability and efficiency. Crosslinking effectively complements orthogonal solvent strategies [10]; however, this section will focus on solvent orthogonality, which is harder to predict, and depends on specific material modifications and post-treatments.

### 6.2. Solvent Compatibility Matrix

To evaluate materials for each OLED layer, it is very important to choose adjacent materials whose solubility properties are not similar. To illustrate this, a solvent compatibility matrix is introduced. This matrix will analyze the compatibility of the most common solvents used in each layer, offering a quick reference for validating potential designs. Solvent comparison is based on Hansen solubility parameters.

Hansen solubility parameters are an experimental method used to estimate how close solvents are by calculating their Hansen distance. This provides a good approximation of solvent orthogonality.

In order to calculate the Hansen distances, we need the bond parameters for each solvent:

- $\delta_{D}$, the dispersion bonds.
- $\delta_{P}$, the polar bonds.
- $\delta_{H}$, the hydrogen bonds.

Then, when comparing two solvents, the following formula is used to calculate the Hansen Distance:

(1) $$R_{a}=\sqrt{4{\left(\delta_{D_{1}}-\delta_{D_{2}}\right)}^{2}+{\left(\delta_{P_{1}}-\delta_{P_{2}}\right)}^{2}+{\left(\delta_{H_{1}}-\delta_{H_{2}}\right)}^{2}}$$

According to the HSP Application note 8, H. Yamamoto, “roughly speaking, HSP distance are lower than 8, that solvent dissolve very well. And the distance are larger than 10 it will not dissolve so much”. Intermediate distances are therefore ambiguous. It is also important to note that solvents are evaluated in their pure form, with no crosslinking taken into consideration.

Therefore:

- $R_{a}<8$ means that adjacent solvents are incompatible (not orthogonal).
- $8\leq R_{a}\leq 10$ means that adjacent solvents have moderate compatibility (uncertain).
- $R_{a}>10$ means that adjacent solvents are compatible (orthogonal).

A full comparison of all solvents used in solution-processed OLED fabrication would be impractical, so the matrix focuses on the most commonly used materials and their typical solvents found in the literature. It is important to emphasize that this matrix serves as an example of how solvent selection can guide the formulation of solution-processed OLEDs. However, the choice of solvents depends on multiple factors, including the deposition method, ink formulation and orthogonality requirements. As a result, the solvents presented here may not be the optimal choices for every design or fabrication process. Only silver nanoparticles are considered as cathode materials, reflecting the current scarcity of viable solution-processed cathodes. The evaluated materials can be found in Table 7.

The following Table 8, Table 9, Table 10, Table 11 and Table 12,when cross-evaluated with which materials dissolve in which solvents, can help produce a list of possible OLED layer stacks that respect solvent miscibility constraints.

### 6.3. Analysis of a Design

Zhao et al. [25] proposed a solution-processed OLED design that combines the HTL and EML layers to simplify fabrication.

Their reported design as: “Solution-processed OLEDs were investigated with the configuration (Figure 2) of: indium tin oxide (ITO)/poly(ethylenedioxythiophene):poly(styrenesulfonate) (PEDOT:PSS) (30 nm)/PVK (35 nm)/PO-T2T (55 nm)/lithium fluoride (LiF) (0.8 nm)/aluminum (Al) (80 nm)”.

While the authors describe their device as “all solution-processed”, a closer examination of the fabrication process reveals that the cathode (Al), EIL (LiF) and ETL (PO-T2T) were deposited using vacuum thermal evaporation: “50 nm of PO-T2T, 1 nm of LiF and 100 nm of aluminium were thermally evaporated in high vacuum ($10^{-6}$ mbar) at the rates of 1.0, 0.1 and 10.0 Å s^−1^, respectively” [25]. This indicates that the device is in fact a hybrid design with only the HIL (PEDOT:PSS) and HTL/EML (PVK) being solution processed. For the purpose of this analysis, we will focus only on the solution-processed layers, ignoring the anode and cathode, as shown in Table 13.

Referring to the Hansen solubility parameters (HSP) between each adjacent layer, we obtain values listed in Table 14:

We can now create an HSP Matrix using the HSP distance formula:

(2) $$R_{a}=\sqrt{4{\left(\delta_{D_{1}}-\delta_{D_{2}}\right)}^{2}+{\left(\delta_{P_{1}}-\delta_{P_{2}}\right)}^{2}+{\left(\delta_{H_{1}}-\delta_{H_{2}}\right)}^{2}}$$

According to the HSP Application Note 8 by H. Yamamoto: “roughly speaking, HSP distance are lower than 8, that solvent dissolve very well. And the distance are larger than 10 it will not dissolve so much”.

The calculated $R_{a}$ value of 42.64, seen in Table 15, confirms that the selected solvents exhibit sufficient orthogonality, ensuring that the deposition of the PVK layer (from chlorobenzene) does not dissolve the underlying PEDOT:PSS layer (deposited from water). This value is significantly higher than the threshold of 10, confirming excellent solvent resistance at this interface. The solvent choices for the solution-processed portion of Zhao et al.’s paper [25] are therefore justified.

However, the lack of explicit formulation details, particularly for the PVK used in the EML, introduces uncertainty in analyzing the OLED structure. Without clear documentation, assumptions had to be made regarding the exact solvent formulation.

### 6.4. Gaps and Limitations in the Solvent Selection Literature

Although solvent choice is fundamental to the fabrication of solution-processed OLEDs, explicit justification for solvent selection is often absent from the literature. Solvent orthogonality—essential for stacking multiple solution-processed layers—is frequently assumed rather than demonstrated. Device functionality is commonly treated as implicit proof of compatibility, with little discussion of why specific solvents were chosen or how potential interlayer interactions were addressed.

While some studies acknowledge the importance of orthogonal solvent systems, this is rarely supported by comparative analyses or detailed formulation rationale. Reported solvents are often listed without mention of alternative options, rejected candidates, or the underlying decision-making process. Key parameters such as solubility profiles, drying dynamics, and boiling points are seldom discussed in depth, despite their strong influence on film formation, morphology, and interlayer stability.

Scalability also remains underexplored. Chlorinated solvents, though prevalent in laboratory studies, are unsuitable for industrial manufacturing [4], yet greener or more scalable alternatives are rarely evaluated or compared [72]. In certain cases, intentional interlayer mixing is used as a design strategy [10], but these approaches are seldom contrasted with fully orthogonal stacks or framed in terms of process trade-offs.

Ink formulations present another reporting gap: viscosity, additives, surface tension, and dispersion stability—parameters that directly influence deposition quality and reproducibility—are often omitted. This leaves readers without the context needed to reproduce results or evaluate the robustness of the process.

The solvent-compatibility matrix presented in this section demonstrates that structured documentation of solvent selection is both feasible and informative, even without experimental validation. When solvent properties are known or measurable, their selection should be explicitly justified. Improved transparency in this regard would significantly enhance clarity, reproducibility, and comparability across the field.

## 7. Thermal Considerations

In addition to material and solvent selection, thermal constraints have to be considered. They significantly affect both fabrication and device stability. Unlike vacuum-processed OLEDs, where sublimation and decomposition temperature are the main thermal concerns, solution-processed OLEDs face additional challenges mainly due to the use of liquid solvents. Beyond solvent interactions, annealing conditions and the thermal stability of the materials used in the OLED stack must also be taken into account.

### 7.1. Boiling Points

Solvent boiling points and evaporation rates strongly influence processing conditions and must be carefully considered. Solvents with low boiling points will evaporate too quickly, leading to non-uniform films and poor layer adhesion [10]. High boiling point solvents can prolong drying times, increasing the risk of damaging underlying layers through extended heating and solvent exposure [31]. However, they may be beneficial in specific applications such as slot die coating [31], where maintaining a wetted coating head is preferred. Evaporation rate also influences film morphology [19]. Rapid solvent evaporation can lead to metastable structures, which may improve or hinder performance depending on charge transport properties [14].

### 7.2. Degradation Limits

Organic materials typically degrade between 300 °C and 450 °C [5], which limits the maximum processing temperatures of the materials before thermal degradation occurs. Solution-processed materials must therefore remain stable at lower temperatures to avoid degradation, requiring processing methods such as thermal annealing to be optimized within those thermal limits.

### 7.3. Glass Transition Temperature ($T_{g}$)

The glass transition temperature $T_{g}$ is a key factor in determining film stability, though it does not always impose strict processing limits. Materials with higher $T_{g}$ values are preferred, as they help prevent phase transitions during fabrication [5]. Annealing below $T_{g}$ aids in solvent removal and can improve film uniformity. Annealing above $T_{g}$ may improve molecular packing but increases the risk of intermixing or recrystallization [5]. PEDOT:PSS, for instance, remains stable even above its $T_{g}$, especially in post-treated films [73].

### 7.4. Annealing

Annealing is used to improve film morphology and charge transport properties [18]. However, it must remain within the thermal limits of the OLED stack. Some materials require specific annealing temperatures for crosslinking to enhance solvent resistance, but these temperatures may degrade other layers if thermal compatibility is not ensured. For example, PEDOT:PSS has been shown to become water-stable after a short high-temperature baking [74]. Annealing must take into account not just the adjacent layer, but all underlying layers, as it may lead to intermixing or degradation of the previously deposited layers.

### 7.5. Drying and Film Formation

Drying dynamics strongly influence film morphology and uniformity. Solvent evaporation rate, surface tension and ink formulation parameters directly impact final film quality [8]. A commonly observed issue, especially in inkjet printing, is the coffee-ring effect, where solvent flows outward during drying, leaving a thicker deposit at the edges and compromising uniformity [8,12]. This effect can be mitigated by selecting appropriate solvents, heating the substrate or applying surface treatments [8].

### 7.6. Sintering

Sintering is used to convert particulate and nanoparticle-based inks into uniform, conductive films [38]. It helps reduce resistance and improve particle connectivity by removing dispersants and decomposing precursors. Methods used include thermal (furnace, hotplate), laser, flashlamp, microwave, plasma, electrical, and chemical sintering, each defined by specific time and process parameters [75]. For example, Ag-NPs can be sintered in a furnace between 50 to 400 °C for up to 30 min, demonstrating a wide processing window that depends on application requirements [38]. Particle size and substrate thermal conductivity directly influence the result. Over-sintering may lead to pore or crack formations, rupture or delamination [75].

### 7.7. Gaps and Limitations in the Thermal Stability Literature

Thermal stability in solution-processed OLEDs remains insufficiently characterized across most published work. Although the glass transition temperature ($T_{g}$) is widely recognized as an important parameter, its relationship to film morphology, interfacial structure, and long-term device performance is still poorly understood. Annealing protocols are often described only qualitatively, and optimal thermal budgets for complete multilayer stacks remain largely unexplored. Similarly, the thermal behavior of interfaces—along with the effects of residual solvents trapped within films—is increasingly acknowledged as a performance-limiting factor but remains only sparsely quantified across different materials and layer thicknesses. As a result, managing thermal limits and heat dissipation during processing continues to be a major constraint in solution-processed OLED fabrication.

A structured, stack-based thermal budget—reporting temperatures, durations, and ambient conditions applied after each deposition step, and constrained by the most heat-sensitive underlying layer—would offer a more systematic way to document and compare device architectures. Quantifying these variables and evaluating them against known thermal limits would support more rigorous material screening, improved layer compatibility assessment, and clearer identification of failure mechanisms.

While meaningful progress has been made in recognizing thermal challenges during both fabrication and operation, the field would benefit from more quantitative, standardized reporting. The absence of consistent layer-by-layer thermal documentation remains a significant barrier to reproducibility and limits the development of optimized, thermally robust solution-processed OLED stacks.

## 8. Monte Carlo Optimization

To systematically generate viable OLED stack configurations under real or approximated fabrication constraints, a Monte Carlo optimization framework was developed. By randomly sampling and mutating candidate stacks, the algorithm explores the design possibilities for viable OLED configurations. Mutations included changing individual materials or solvents with a tunable probability.

Each stack was scored using a weighted function that incorporated work function alignment, thermal compatibility, solvent orthogonality, materials reuse, and completeness. Hard constraints such as solvent compatibility with the materials were applied up front. The score then allowed prioritization of higher-scoring candidates based on optimization targets. Although thermal compatibility was included, precise annealing temperatures were generally unavailable in the literature, reflecting a gap in the available data. Approximate temperature ranges were instead used.

As the implementation follows a standard Monte Carlo approach, the code is not included. A simplified flowchart presents the logic path, while the focus is placed on the scoring logic and result viability. It is also important to note that while HSP parameters were judged very strictly in their corresponding section, a relaxed threshold was used in this framework. In real-life processing, materials and their solvents are often processed beyond their reported thermal limits or combined with adjacent solvents despite limited orthogonality. This also aims to take into account material doping and solvent crosslinking, which cannot be easily quantified into strict numerical data.

Stacks below a defined threshold were discarded during selection but retained in the graphs to illustrate result distribution. The process then produced layer-by-layer scoring breakdowns to allow visualization of convergence and overall distribution over time.

As a proof of concept rather than an exhaustive search, not all materials listed in the literature review were included. Those with sufficient characterization data, known solvent compatibility and diversity were used. For instance, Mg-Ag cathodes, though covered in the materials section, were excluded due to redundancy with Ag-NPs and limited formulation data.

### 8.1. Optimization Workflow Overview

**Figure 5:**
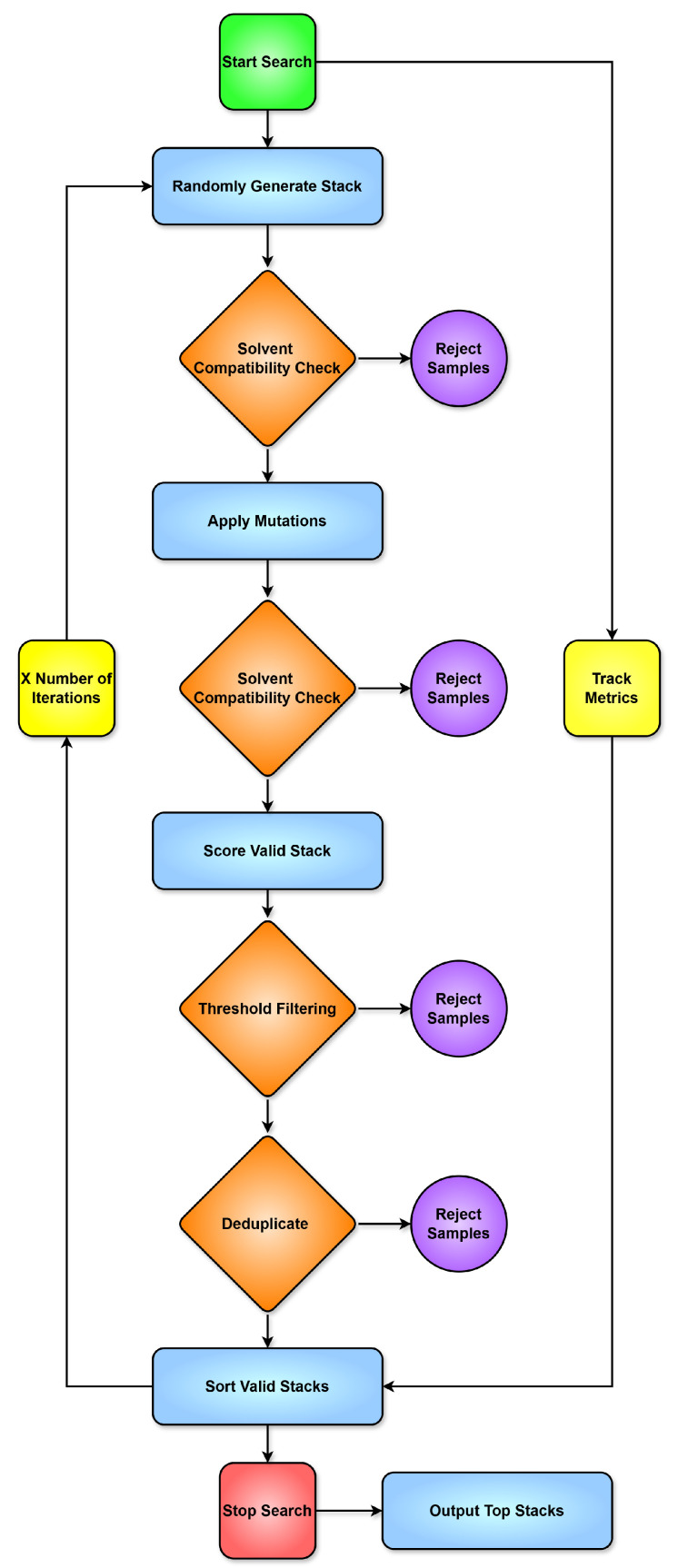
Optimization logic workflow.

To illustrate the logic of the Monte Carlo search, a simplified flowchart of the optimization process is shown in Figure 5. The process begins with a random stack generation, where materials and solvents are assigned. HSP parameters are used to check for compatibility and to make the first rejection pass. Mutations are then applied, followed by a second compatibility check on the mutated stack. If it passes, the stack is scored using a weighted set of criteria. A final filtering step is applied using a score threshold. Convergence is tracked during the search, and duplicate stacks are removed from the results.

### 8.2. Scoring Parameters

The scoring configurations used during the Monte Carlo search are presented in Table 16 and Table 17. Each parameter rewards desirable properties or penalizes violations based on fabrication constraints, material compatibility or electrical performance. Hard constraints are applied prior to scoring, while the weighted components influence final stack selection. Scoring values were based on theoretical considerations, rough estimates, and trends observed in published stacks. For example, solvent incompatibility is not strictly binary in practice. This is reflected in the modest bonus applied for non-adjacent solvent reuse. The triple use penalty targets stacks where PEDOT:PSS is reused for the Anode, HIL and HTL, a choice that is economical in terms of stack complexity, but one that significantly limits stack diversity. It therefore forces the algorithm to explore alternative HTLs once the anode and HIL are already fixed with PEDOT:PSS. It is important to note that this section serves as a proof of concept. Intervals, values, and scores have been estimated and defined by the author. The scored stacks should be interpreted as such.

Due to the limited reporting of processing temperatures in the literature, the following categories were derived from manufacturer datasheets and available annealing temperature data.

### 8.3. Limitations and Future Work

This section, along with Section 9, serve as a proof of concept. We used neutral weights, heuristic inputs, and placeholder values where needed. Stack rankings are driven by material properties and HSP-based orthogonality values, helping assess the validity of the method. The material pool was limited, with cathode selection remaining the primary bottleneck.

Future work includes building a structured dataset that links materials, solvents, and process constraints. This would allow the same framework to produce results more closely aligned with each material’s physical constraints.

## 9. OLED Layer Stack Configuration

This section brings together the layer selection constraints discussed in the materials and solvent sections, along with the Monte Carlo optimization framework, to generate viable OLED stacks. Building on the solvent compatibility matrix, thermal processing limitations are introduced, albeit in simplified form. Because precise annealing temperatures are rarely reported, materials are categorized by approximate processing ranges rather than fixed values. This enables an approximate evaluation of thermal constraints on stack feasibility and selection. The scoring weight assigned to thermal constraints is reduced to reflect this limitation. The resulting combinations aim to reflect realistic fabrication conditions by avoiding strict binary decisions when possible. For example, selection using Hansen solubility parameters is loosened to reflect solvent variability introduced during laboratory processing. This also aims to account for doping and solvent crosslinking, which could not be quantified due to the absence of reported numerical data.

### 9.1. Controlling Diversity in Stack Selection

Given the high mutation rate and large number of scoring parameters applied to a small dataset of materials and solvents, many high scoring stacks tend to be similar. To better control variability in the output set, a crossfade parameter was introduced. This parameter balances material diversity and solvent diversity in the accepted results. At a crossfade value of 0%, the algorithm prioritizes distinct material combinations, penalizing and rejecting stacks that reuse the same materials even if solvents differ. At 100%, the opposite occurs and stacks may reuse identical materials as long as the solvents differ significantly. A value of 50% produces a more balanced selection, though the over-representation of solvents in the dataset still skews results towards solvent variability.

This mechanism could serve as a post-scoring diversity filter to balance material diversity and fabrication flexibility through solvent variation, depending on the focus.

Table 18 shows that prioritizing material diversity significantly reduces the number of accepted stacks, while emphasizing solvent variation yields results comparable to the balanced setting. In the following section, representative stacks will be presented to illustrate the range of configurations produced using the most impactful optimization settings.

### 9.2. Optimization Results

The Monte Carlo optimization generated a broad pool of OLED stacks that satisfied the combined fabrication constraints, including solvent compatibility, thermal processing range, work function alignment and electrical properties. Although thousands of configurations were created, only a subset passed the hard constraints, crossfade diversity filters and score threshold. The distribution of the scores and the structure of the high-scoring stacks offer insight into how theoretical constraints interact with practical design solutions. In this section, we present representative stacks, highlight recurring patterns and analyze how different scoring components influenced selection. Note that a “shared” solvent entry indicates layers deposited simultaneously using the same solvent. Higher scores represent a better match with the defined scoring parameters, but do not imply a better performance.

**Figure 6:**
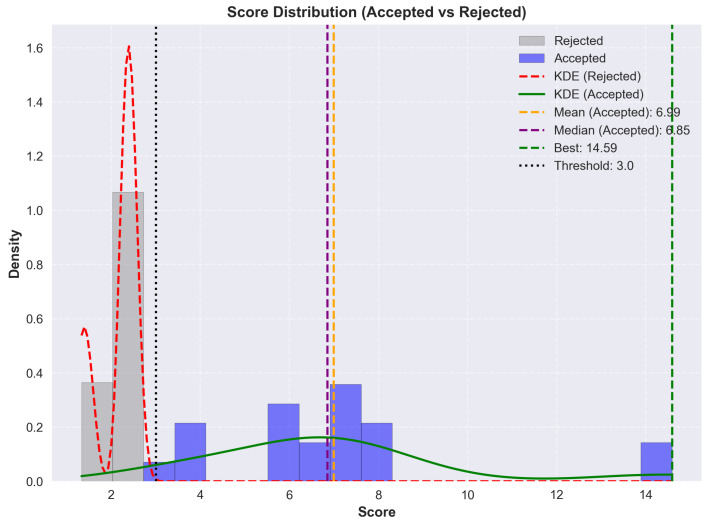
Distribution of OLED stacks—minimum score threshold of 3, crossfade of 10%.

**Figure 7:**
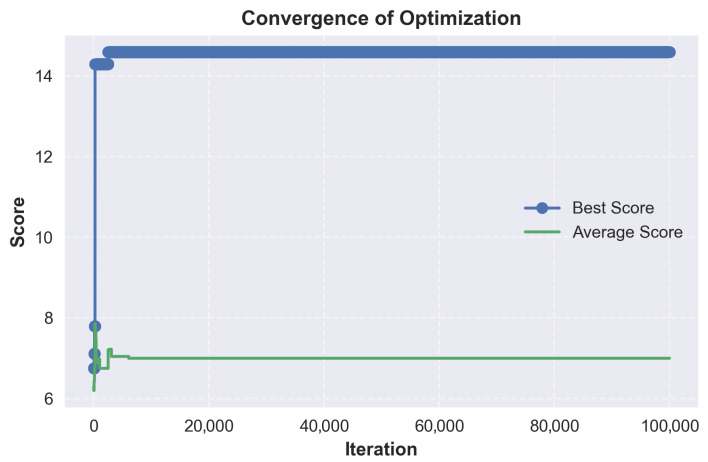
Convergence of OLED stacks—minimum score threshold of 3, crossfade of 10%.

The impact of prioritizing material diversity is visualized in Figure 6 and Figure 7. At a material/solvent crossfade of 10% and a minimum score threshold of 3.0, the accepted results are hovering around 20 stacks, which showcases the limited material variability when prioritizing diverse stacks. The accepted stacks form a cluster around a score of 7, but we still see configurations that have a score of almost 15. The majority of stacks have fallen below the score threshold. Convergence is reached early, with the best score appearing in the first few thousand iterations and staying constant. The average score plateaus quickly, which indicates that viable stacks are primarily discovered early and mutations mainly affect solvent variants. This confirms that while solvent flexibility introduces useful diversity, a crossfade skewed towards material variability will heavily impact the number of viable stacks.

As illustrated in Table 19, applying the strongest bias towards material variety, we obtain two highly scored stacks, followed by a sharp drop in score. We also note that while material variety was prioritized, the higher scoring stacks still use PEDOT:PSS as the first two or three layers.

**Figure 8:**
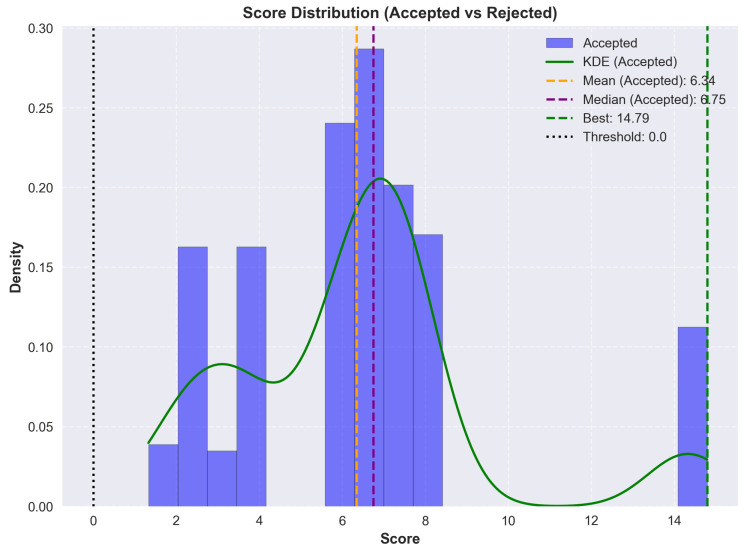
Distribution of OLED stacks—minimum score threshold of 0, crossfade of 45%.

**Figure 9:**
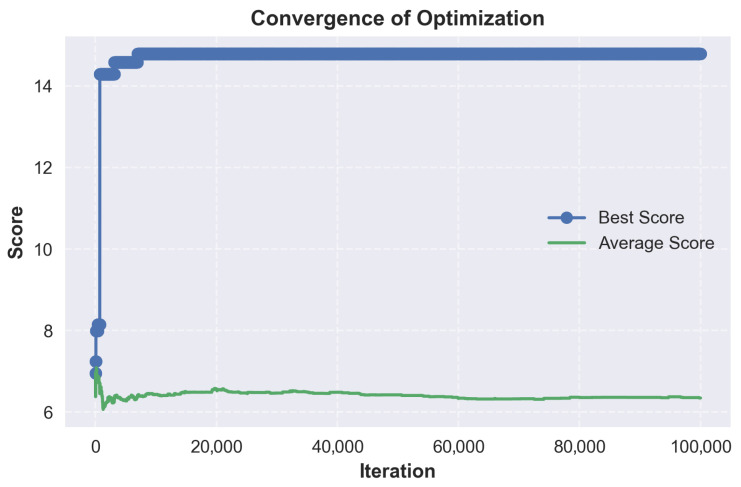
Convergence of OLED stacks—minimum score threshold of 0, crossfade of 45%.

The broader search space exploration enabled by a balanced crossfade is depicted in Figure 8 and Figure 9. At a material/solvent crossfade of 45% and a minimum score threshold of 0, the optimizer explores the full search space without early rejections. The distribution forms a clearer peak around a score of 6.3, with the majority of accepted stacks between 5 and 8. A secondary peak near 15 indicates the presence of high-performing configurations. Convergence is again fast, but the average score fluctuates more, which is caused by the lack of score thresholding and the mutation. This run confirms that a balanced material/solvent crossfade allows a broader exploration of viable stacks.

As illustrated in Table 20, with a balanced optimization, we note that the top-scoring stacks all use the same material configuration, with only the solvents showing variability. Most variations occur in the solvent choice for the ETL and HIL, where alcohol-based solvents are interchanged without affecting stack viability. This suggests that ZnO nanoparticles and PEDOT:PSS can tolerate a better range of polar solvents.

**Figure 10:**
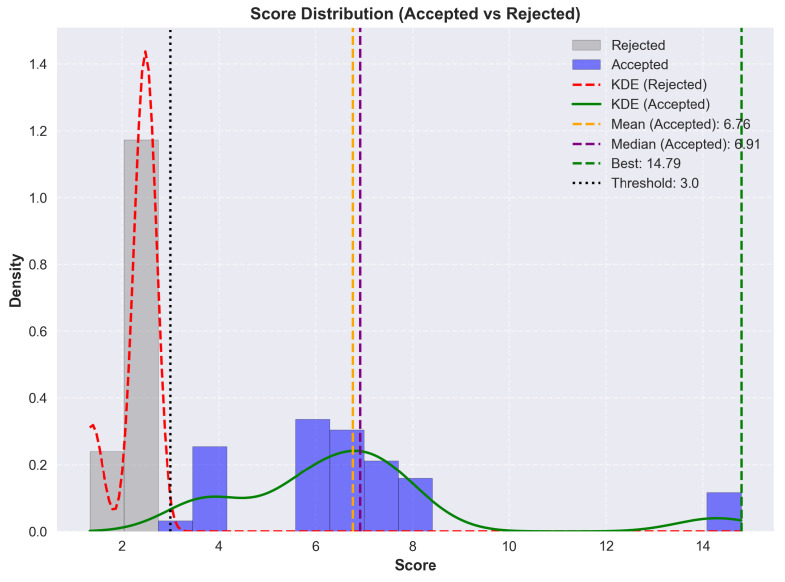
Distribution of OLED stacks—minimum score threshold of 3, crossfade of 100%.

**Figure 11:**
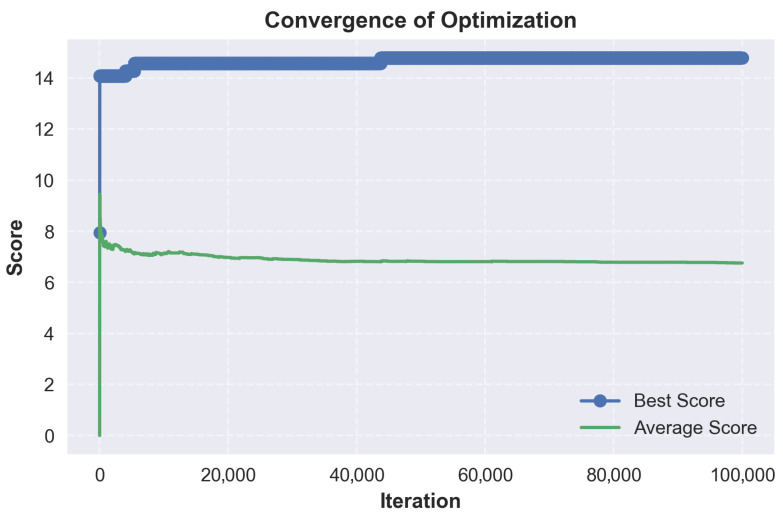
Convergence of OLED stacks—minimum score threshold of 3, crossfade of 100%.

The impact of maximizing solvent diversity is shown in Figure 10 and Figure 11. At a material/solvent crossfade of 100% and a minimum score threshold of 3.0, the optimizer favors solvent variability exclusively. The accepted scores are similar to the balanced run, and scores are clustered between 6 and 8, with outlying scores around 15. Convergence is reached quickly, but the average score can be seen declining over time. This can be explained by the optimizer exploring different solvent variations, leading to marginal or lower scores.

As illustrated in Table 21, with the strongest bias towards solvent variety, we obtain results that are virtually identical to the balanced run. All top performing stacks rely on the same material configuration with the only differences being in the assigned solvents. While solvent variability enables the generation of stack variants, this helps confirm that material choice is a stronger factor in achieving high performance.

### 9.3. Analysis

From the generated results, a few common threads consistently emerge. PEDOT:PSS appears in every top-scoring stack as both the anode and HIL, confirming its reliability as a dual-function material. Its vast solvent compatibility and suitable work function make it the default choice for hole injection. The optimizer occasionally selected it for triple use, as the anode, HIL and HTL. This was explicitly disabled to increase stack diversity by revealing a broader range of viable materials. In both the balanced and solvent biases cases, the top stacks are nearly identical. This similarity suggests that while solvent diversity introduces flexibility, material choice remains the determining factor in stack viability.

TAPC appears as the dominant HTL across nearly all viable stacks, and PVK is consistently used for the EML. This suggests a strong compatibility between TAPC and PVK. ZnO nanoparticles are likewise selected in almost every case for the ETL.

Cathode selection remains the most constrained, with only silver nanoparticles appearing across all accepted configurations. No other material satisfies the combined requirements of solution processability, work function and orthogonal solvent use. The cathode therefore constitutes the bottleneck in fully solution-processed OLEDs.

Despite these constraints, almost 400 stacks were generated on the least restrictive settings. Compared to published designs, the results show strong overlap in early layers, with PEDOT:PSS and PVK appearing consistently in both cases. However, the optimizer systematically selects TAPC as the HTL and ZnO nanoparticles as the ETL, which are materials that are less common in the literature subset but offer good solvent compatibility. The largest divergence is in the cathode, as expected. While most published designs rely on evaporated LiF/Al or Ba/Al, few explore silver, and even fewer consider solution processable cathodes [75].

Finally, as an additional proof of concept, we can compare our results with working OLED stacks from the literature. For instance, as shown in Table 22, the method reproduced the same ETL/EML/HTL stack from Zhao et al. [25], which was analyzed previously.

To further validate the method’s capability in identifying fully solution-processed architectures, we compared the results against the work of Amruth et al. [31], shown in Table 23.

In this design, all organic and polymer layers were successfully deposited using slot die coating. However, the Ag cathode required vacuum thermal evaporation. This highlights the consistent bottleneck in the field and shows how thermal evaporation still is relied upon to avoid the challenges of solution-processing the cathode.

Table 24 presents a structurally equivalent stack generated by the Monte Carlo method.

The primary difference is the use of Super Yellow and MEH-PPV. However, both are PPV derivatives and common solution-processable emissive polymers. They also share the same solubility profile, making them functionally equivalent for this comparison.

It is worth noting that the tool was configured with a limited library of materials and solvents and therefore was permitted to select repeating materials for the anode, HIL, and HTL. Allowing it here demonstrates that the algorithm can correctly reproduce viable experimental designs that rely on simplified material sets. The tool also identified an alternative stack using TAPC dissolved in THF for the HTL. However, the PEDOT:PSS-based configuration was selected here for its direct structural similarity to the experimental reference.

This Monte Carlo selection method, given a large enough dataset and improved experimental reporting, could help identify viable yet understudied material combinations.

## 10. Discussion

Across the literature on solution-processed OLEDs, a consistent limitation is the incomplete reporting of materials, formulations, and processing parameters. While device performance is almost always emphasized, the underlying fabrication details—critical for reproducibility—are often missing or only partially disclosed. Solvents, additives, material grades, and supplier information are rarely specified with precision. Even widely used materials such as PEDOT:PSS or PVK appear in multiple commercially available variants with markedly different conductivities, viscosities, and stabilizing additives, yet publications frequently omit which formulation was used or how it was processed. This lack of detail significantly constrains the ability of other researchers to replicate or systematically build upon existing work.

Similar gaps appear in the reporting of surface treatments, post-deposition annealing conditions, and layer-by-layer fabrication steps. Stack diagrams commonly illustrate the overall device architecture, but essential information—layer thicknesses, crosslinking conditions, deposition sequences, or the extent of interlayer mixing—is inconsistently provided. Even when interlayer diffusion is acknowledged as a potential challenge, only a few studies quantify it, leaving large uncertainties in interpreting device behavior.

Electrical characterization practices also show variation. Although key performance metrics are widely reported, the testing conditions under which these values were obtained are often absent. Lifetime measurements, in particular, lack standardization, and degradation mechanisms are frequently modeled empirically without probing underlying physical or chemical processes [5]. As Zhang et al. emphasize, without clear testing standards, results reported across different studies cannot be meaningfully compared [14].

Reproducibility remains another largely unaddressed concern. Few publications provide information on process tolerance, failed attempts, or device-to-device variability—factors essential for evaluating the robustness and scalability of a proposed method. As a result, newcomers to the field face an opaque design space. The absence of rationale behind material and solvent choices makes it difficult to establish a consistent starting point or understand why specific decisions were made.

These challenges motivated the development of the Monte Carlo–based optimization framework presented in this work. Rather than seeking the absolute best-performing OLED stack, the goal was to demonstrate how a systematic, data-driven methodology could be used to explore design choices and evaluate layer compatibility based on known material properties and fabrication constraints. This framework serves as a proof of concept for how OLED stack design can be formalized, provided that the underlying empirical data is sufficiently documented.

In the long term, this work points toward a broader opportunity: the creation of a standardized, collaborative database of OLED materials, formulations, process parameters, and device outcomes. Such a resource, coupled with computational tools for evaluating compatibility and predicting performance, could greatly improve transparency, reproducibility, and methodological consistency across the field. Recording not only results but also the reasoning behind design choices would help accelerate cumulative progress in this rapidly evolving area.

## 11. Conclusions

This work has presented a comprehensive review and critical evaluation of materials and fabrication strategies used in solution-processed OLEDs. Despite significant advances in printable and scalable device architectures, progress is hindered by persistent gaps in documentation—particularly regarding solvent systems, material formulations, and processing conditions. The limited reporting of design rationales further complicates reproducibility and slows the accumulation of systematic knowledge in the field.

To address these challenges, a Monte Carlo-based optimization framework was introduced as a proof of concept for formalizing OLED stack design. Rather than optimizing for peak performance, the framework demonstrates how material parameters, fabrication constraints, and approximate models can be integrated into a structured decision-making process. This approach highlights the value of rigorous, quantitative methodologies in guiding layer selection and evaluating compatibility.

Overall, the study underscores the need for a standardized analytical framework and more comprehensive reporting practices in solution-processed OLED research. A collaborative, community-driven database documenting formulations, processing parameters, and design rationales could provide a foundation for more transparent, reproducible, and iterative development of OLED technologies. Such efforts would not only help researchers navigate an increasingly complex materials landscape but also support the advancement of reliable, scalable, and high-performance solution-processed OLED devices.

## Figures and Tables

**Table 1:** Comparison of OLED, LED, LCD, and QLED Technologies.

Feature	OLED [5]	LED [6]	LCD [7]	QLED [8]
Material	Organic semiconductors	Inorganic semiconductors	Liquid crystals	Quantum dots with LCD backlight
Light Generation	Self-emissive: organic layers emit light when current passes through	Light emitted directly from semiconductor diodes	Uses LED backlight and liquid-crystal shutters to control brightness	Uses quantum dots to enhance LED backlight
Applications	Displays, lighting, flexible electronics	Displays, lighting, indicators	Displays (TVs, monitors, mobile devices)	High-end displays, TVs
Key Strengths	High contrast, deep blacks, self-emissive, fast response, flexible form factor	High brightness, long lifespan, energy-efficient	Mature technology, affordable, good brightness	Better colors than standard LCD, higher brightness than OLED
Key Limitations	Higher cost, shorter lifespan, risk of burn-in, sensitive to moisture	Not self-emissive, limited color accuracy	Requires backlight, lower contrast, slower response time	More expensive than LCD, still requires backlight

**Table 3:** Summary of recent solution-processed fluorescent OLED performance.

Emitter (Host)	Deposition Method	Device Structure	Max EQE (%)	Max Eff. (cd/A)	$\lambda_{\mathrm{EL}}$ (nm)	Ref.
2DPATrxSO2 (CBP)	Spin-coating	ITO/PEDOT:PSS/ CBP:2DPATrxSO2/ TPBi/LiF/Al	10.6	11	488	[40]
F8BT (TFB)	Spin-coating	ITO/PEDOT:PSS/ TFB/F8BT/ poly(DDA)TFSI/Al	9.0	30.1	540	[1]
Pt2a (mCP)	Spin-coating	ITO/PEDOT:PSS/ mCP:Pt2a/DPEPO/ TmPyPB/Liq/Al	8.7	14.3	614	[41]
H-DPPN (TCTA)	Spin-coating	ITO/PEDOT:PSS/ TCTA:H-DPPN/ TPBi/LiF/Al	4.0	11.8	570	[42]
BUBD-1 (MADN)	Blade-coating	ITO/PEDOT:PSS/ TFB/NPB:MADN:BUBD-1/ Alq3/LiF/Al	2.57	5.68	464	[43]

**Table 4:** Summary of recent solution-processed phosphorescent OLED performance.

Emitter (Host)	Deposition Method	Device Structure	Max EQE (%)	Max Eff. (cd/A)	$\lambda_{\mathrm{EL}}$ (nm)	Ref.
Bt2Ir(acac) (TCTA:3PTPS)	Spin-coating	ITO/GraHIL/ TCTA:3PTPS:Bt2Ir-(acac)/TPBi/LiF/Al	35.5	97.5	563	[2]
Ir(ppy)3 (TCTA:2PTPS)	Spin-coating	ITO/GraHIL/ TCTA:2PTPS:Ir(ppy)3 /TPBi/LiF/Al	29.0	101.5	514	[2]
mer-Ir(CF3pbp)3 (TSPO1)	Spin-coating	ITO/PEDOT:PSS/ TSPO1:mer-Ir(CF3pbp)3/ TSPO1/TPBi/LiF/Al	21.2	7.7	423	[44]
Ir(Th-PQ)2 (TCTA:TPBi)	Spin-coating	ITO/GraHIL/ TCTA:TPBi:Ir(Th-PQ)2/ TPBi/LiF/Al	21.0	26	612	[45]
Ir3 (TCTA:TPBi)	Spin-coating	ITO/m-PEDOT:PSS/ TCTA:TPBi:Ir3/TPBi/LiF/Al	24.08	91.98	523	[46]

**Table 5:** Summary of recent solution-processed TADF OLED performance.

Emitter (Host)	Deposition Method	Device Structure	Max EQE (%)	Max Eff. (cd/A)	$\lambda_{\mathrm{EL}}$ (nm)	Ref.
4CzIPN (CBP)	Spin-coating	ITO/Buf-HIL/ CBP:4CzIPN/TPBi/ LiF/Al	24	73	511	[47]
2SPAC-DBP-2tBuCz (TCTA:26DCzPPy)	Spin-coating	ITO/PEDOT:PSS/ 2SPAC-DBP-2tBuCz: TCTA:26DCzPPy/ B4PyPPM/LiF/Al	23.7	38.6	583	[48]
DMeCzIPN (PVK/mCP)	Spin-coating	ITO/PEDOT:PSS/PVK/ DMeCzIPN:mCP/ DPEPO/TmPyPB/ LiF/Al	21.6	44.3	478	[49]
ODTBPZ-DPXZ (TCTA)	Spin-coating	ITO/PEDOT:PSS/ TCTA:ODTBPZ-DPXZ/ TmPyPB/LiF/Al	18.5	31.0	612	[50]
BCzBN (SBA-2DPS—Host-free ink)	Inkjet Printing	ITO/PEDOT:PSS/ mCP:PVK/ SBA-2DPS:BCzBN/ DPEPO/TmPyPB/Liq/Al	10.1	18.7	488	[3]

**Table 6:** Material properties. HOMO values are used as approximations for work function where direct measurements are unavailable. N/A: Not Available.

Layer	Material	Work Function (eV)	Conductivity (S· cm^−1^)
Anode	PEDOT:PSS	4.7–5.4 [53]	$10^{-4}$–$10^{3}$ [54]
HIL	PEDOT:PSS	4.7–5.4 [53]	$10^{-4}$–$10^{3}$ [54]
HTL	PEDOT:PSS	4.7–5.4 [53]	$10^{-4}$–$10^{3}$ [54]
	PVK	5.65 [55]	$2\cdot 10^{-8}$ [56]
	TPD	5.5 [57]	N/A
	TAPC	5.5 [58]	N/A
EML	F8:F8BT	5.9 [59]	$10^{-3}$
	PVK	5.65 [55]	$2\cdot 10^{-8}$ [56]
	MEH-PPV	5.3 [60]	$2.47\cdot 10^{-11}$ [61]
ETL	TPBi	6.2 [62]	N/A
	PFN	4.1 [63]	N/A
	PFN-Br	3.86 [64]	$7\cdot 10^{-7}$ [64]
	ZnO nanoparticles	4.68 [65]	$1.14\cdot 10^{-3}$ [66]
	PO-T2T	7.6 [67]	N/A
Cathode	Ag-NPs	5.29–5.53 [68]	up to $6.6\cdot 10^{4}$

**Table 7:** Solution-processed OLED materials and their solvents/dispersion materials.

Layer	Material	Solvent
Anode	PEDOT:PSS	Water, Ethanol, Isopropanol [24]
HIL	PEDOT:PSS	Water, Ethanol, Isopropanol [24]
HTL	PEDOT:PSS	Water, Ethanol, Isopropanol [24]
	PVK	o-Xylene, Chloroform, Chlorobenzene, Dichlorobenzene [69]
	TPD	Chlorobenzene [24]
	TAPC	THF, Chloroform [58]
EML	F8:F8BT	Chlorobenzene, Toluene [59]
	PVK	o-Xylene, Chloroform, Chlorobenzene, Dichlorobenzene [69]
	MEH-PPV	Toluene, Chlorobenzene [60]
ETL/EIL	TPBi	Chloroform, Chlorobenzene, Toluene [62]
	PFN	Water, Methanol, 1-butanol (with acetic acid) [70]
	PFN-Br	DMF, DMSO, Ethanol, Methanol, Isopropanol mixture, Water [37]
	ZnO NPs (dispersed)	Methanol, Ethanol, Isopropanol [24]
	PO-T2T	Methanol, Ethanol, Isopropanol [25]
Cathode	Ag-NPs (dispersed)	Isopropanol, Ethanol, Water [71]

**Table 8:** Solvent compatibility matrix for anode/HIL. Background colors indicate HSP-based orthogonality: green denotes high compatibility ($R_{a}>10$), and red denotes incompatibility ($R_{a}<8$).

Anode/HIL	Water	Ethanol	Isopropanol
**Water**	0.00	24.11	27.83
**Ethanol**	24.11	0.00	4.04
**Isopropanol**	27.83	4.04	0.00

**Table 9:** Solvent compatibility matrix for HIL/HTL. Background colors indicate HSP-based orthogonality: green denotes high compatibility ($R_{a}>10$), yellow denotes moderate compatibility, and red denotes incompatibility ($R_{a}<8$).

HIL/HTL	Water	Ethanol	Isopropanol	o-Xylene	THF	CB	Dichlorobenzene	Chloroform
**Water**	0.00	24.11	27.83	42.32	36.00	42.64	40.96	39.17
**Ethanol**	24.11	0.00	4.04	18.51	11.98	19.08	17.66	15.37
**Isopropanol**	27.83	4.04	0.00	14.80	8.64	15.86	14.76	11.81

**Table 10:** Solvent compatibility matrix for HTL/EML. Background colors indicate HSP-based orthogonality: green denotes high compatibility ($R_{a}>10$), yellow denotes moderate compatibility, and red denotes incompatibility ($R_{a}<8$).

HTL/EML	Water	Ethanol	Isopropanol	o-Xylene	THF	CB	Dichlorobenzene	Chloroform
**Water**	0.00	24.11	27.83	42.32	36.00	42.64	40.96	39.17
**Ethanol**	24.11	0.00	4.04	18.51	11.98	19.08	17.66	15.37
**Isopropanol**	27.83	4.04	0.00	14.80	8.64	15.86	14.76	11.81
**o-Xylene**	42.32	18.51	14.80	0.00	7.08	4.23	6.00	3.34
**THF**	36.00	11.98	8.64	7.08	0.00	7.57	6.74	4.01
**CB**	42.64	19.08	15.86	4.23	7.57	0.00	2.42	4.57
**Dichlorobenzene**	40.96	17.66	14.76	6.00	6.74	2.42	0.00	4.88
**Chloroform**	39.17	15.37	11.81	3.34	4.01	4.57	4.88	0.00

**Table 11:** Solvent compatibility matrix for EML/ETL. Background colors indicate HSP-based orthogonality: green denotes high compatibility ($R_{a}>10$), and red denotes incompatibility ($R_{a}<8$).

EML/ETL	Toluene	CB	Chloroform	DMF	DMSO	1-Butanol	Isopropanol	Ethanol	Methanol
**o-Xylene**	1.24	4.23	3.34	15.14	17.00	14.01	14.80	18.51	22.92
**Toluene**	0.00	3.52	4.09	15.47	17.11	15.00	15.77	19.41	23.76
**CB**	3.52	0.00	4.57	13.60	14.67	15.11	15.86	19.08	23.17
**Dichlorobenzene**	5.61	2.42	4.88	11.48	12.34	14.06	14.76	17.66	21.55
**Chloroform**	4.09	4.57	0.00	12.01	14.09	11.03	11.81	15.37	19.73

**Table 12:** Solvent compatibility matrix for ETL/cathode. Background colors indicate HSP-based orthogonality: green denotes high compatibility ($R_{a}>10$), yellow denotes moderate compatibility, and red denotes incompatibility ($R_{a}<8$).

ETL/Cathode	DMF	Isopropanol	Ethanol	Water
**Toluene**	15.47	15.77	19.41	43.25
**Chlorobenzene**	13.60	15.86	19.08	42.64
**Chloroform**	12.01	11.81	15.37	39.17
**DMF**	0.00	9.70	9.99	31.42
**DMSO**	3.54	13.10	13.02	32.72
**1-Butanol**	9.60	0.82	4.77	28.54
**Isopropanol**	9.70	0.00	4.04	27.83
**Ethanol**	9.99	4.04	0.00	24.11
**Methanol**	12.00	8.67	4.76	20.45

**Table 13:** Materials and corresponding layers of the OLED structure reported in *Study of All Solution Process Exciplex Organic Light-Emitting Diode* [25].

Material	Layer	Solvent
PEDOT:PSS	HIL	Water
PVK	HTL/EML	Chlorobenzene (implied)

**Table 14:** Hansen solubility parameters of solvents of the OLED structure reported in *Study of All Solution Process Exciplex Organic Light-Emitting Diode* [25].

Solvent	($\delta_{D}$)	($\delta_{P}$)	($\delta_{H}$)	($\delta_{T}$)
Water	15.5	16.0	42.4	47.9
Chlorobenzene	19.0	4.3	2.0	19.6

**Table 15:** HSP distance matrix for the solution-processed layers (HIL and HTL/EML).

	Water	Chlorobenzene
Water	0	42.64
Chlorobenzene	42.64	0

**Table 16:** Scoring configuration used in the Monte Carlo optimization.

Category	Parameter	Value
Work Function	Weight	1.0
	Reward if ΔWF<0.7 eV	+0.3
	Penalty if 0.7<ΔWF<1.0 eV	−0.5
	Penalty if ΔWF>1.5 eV	−1.0
Thermal Constraints	Weight	1.0
	Burn penalty (per violation)	−0.3
	Max total penalty	1.0
	Multi high-T layers penalty	−0.2
	Top layer high-T penalty	−0.2
Conductivity	Weight	1.0
	Minimum required	10S/cm
	Reward if above minimum	up to +0.5
	Penalty if below minimum	up to −0.5
Solvent Reuse	Bonus (non-adjacent, compatible)	+0.3
Material Reuse	Bonus if reused across layers	+0.5
	Triple use penalty (Anode/HIL/HTL)	−0.7
Threshold	Minimum score for retention	0.0

**Table 17:** Processing temperature categories for common OLED materials.

Material	Processing Temperature Category
PEDOT:PSS	High (>200 °C)
PVK	Mid (100–200 °C)
TPD	Low (<100 °C)
TAPC	Mid (100–200 °C)
F8:F8BT	Mid (100–200 °C)
MEH-PPV	Mid (100–200 °C)
TPBi	Mid (100–200 °C)
PFN	Low (<100 °C)
PFN-Br	Mid (100–200 °C)
ZnO Nanoparticles	High (>200 °C)
PO-T2T	Mid (100–200 °C)
Ag-NPs	N/A

**Table 18:** Number of accepted OLED stacks generated at different crossfade values.

Crossfade Setting	Accepted Stack Count
Material Diversity (0%)	24
Balanced (50%)	309
Solvent Diversity (100%)	398

**Table 19:** Top 5 OLED stack configurations generated with high material diversity (crossfade of 10%) and a minimum score threshold of 3.0.

**Stack 1 (Score: 14.59)**		
**Layer**	**Material**	**Solvent**
Anode	PEDOT:PSS	shared
HIL	PEDOT:PSS	Isopropanol
HTL	TAPC	THF
EML	PVK	Chlorobenzene
ETL	ZnO-NPs	Ethanol
Cathode	Ag-NPs	DMF
**Stack 2 (Score: 14.29)**		
**Layer**	**Material**	**Solvent**
Anode	PEDOT:PSS	shared
HIL	PEDOT:PSS	shared
HTL	PEDOT:PSS	Ethanol
EML	PVK	o-Xylene
ETL	ZnO-NPs	Ethanol
Cathode	Ag-NPs	Water
**Stack 3 (Score: 8.15)**		
**Layer**	**Material**	**Solvent**
Anode	PEDOT:PSS	shared
HIL	PEDOT:PSS	shared
HTL	PEDOT:PSS	Water
EML	MEH-PPV	Toluene
ETL	PFN-Br	Water
Cathode	Ag-NPs	Ethanol
**Stack 4 (Score: 7.99)**		
**Layer**	**Material**	**Solvent**
Anode	PEDOT:PSS	shared
HIL	PEDOT:PSS	shared
HTL	PEDOT:PSS	Water
EML	MEH-PPV	Chlorobenzene
ETL	PO-T2T	Ethanol
Cathode	Ag-NPs	Water
**Stack 5 (Score: 7.79)**		
**Layer**	**Material**	**Solvent**
Anode	PEDOT:PSS	shared
HIL	PEDOT:PSS	shared
HTL	PEDOT:PSS	Isopropanol
EML	MEH-PPV	Chlorobenzene
ETL	PFN	1-butanol
Cathode	Ag-NPs	Water

**Table 20:** Top 5 OLED stack configurations generated with balanced diversity (crossfade of 45%) and a minimum score threshold of 0.0.

**Stack 1 (Score: 14.79)**		
**Layer**	**Material**	**Solvent**
Anode	PEDOT:PSS	shared
HIL	PEDOT:PSS	Isopropanol
HTL	TAPC	THF
EML	PVK	o-Xylene
ETL	ZnO-NPs	Isopropanol
Cathode	Ag-NPs	Water
**Stack 2 (Score: 14.79)**		
**Layer**	**Material**	**Solvent**
Anode	PEDOT:PSS	shared
HIL	PEDOT:PSS	Water
HTL	TAPC	THF
EML	PVK	Chlorobenzene
ETL	ZnO-NPs	Methanol
Cathode	Ag-NPs	Water
**Stack 3 (Score: 14.79)**		
**Layer**	**Material**	**Solvent**
Anode	PEDOT:PSS	shared
HIL	PEDOT:PSS	Isopropanol
HTL	TAPC	THF
EML	PVK	o-Xylene
ETL	ZnO-NPs	Methanol
Cathode	Ag-NPs	Isopropanol
**Stack 4 (Score: 14.79)**		
**Layer**	**Material**	**Solvent**
Anode	PEDOT:PSS	shared
HIL	PEDOT:PSS	Ethanol
HTL	TAPC	THF
EML	PVK	o-Xylene
ETL	ZnO-NPs	Ethanol
Cathode	Ag-NPs	Water
**Stack 5 (Score: 14.59)**		
**Layer**	**Material**	**Solvent**
Anode	PEDOT:PSS	shared
HIL	PEDOT:PSS	Ethanol
HTL	TAPC	THF
EML	PVK	o-Xylene
ETL	ZnO-NPs	Isopropanol
Cathode	Ag-NPs	DMF

**Table 21:** Top 5 OLED stack configurations generated with high solvent diversity (crossfade near 100%) and a minimum score threshold of 3.0.

**Stack 1 (Score: 14.79)**		
**Layer**	**Material**	**Solvent**
Anode	PEDOT:PSS	shared
HIL	PEDOT:PSS	Ethanol
HTL	TAPC	THF
EML	PVK	o-Xylene
ETL	ZnO-NPs	Ethanol
Cathode	Ag-NPs	DMF
**Stack 2 (Score: 14.79)**		
**Layer**	**Material**	**Solvent**
Anode	PEDOT:PSS	shared
HIL	PEDOT:PSS	Ethanol
HTL	TAPC	THF
EML	PVK	o-Xylene
ETL	ZnO-NPs	Ethanol
Cathode	Ag-NPs	Water
**Stack 3 (Score: 14.79)**		
**Layer**	**Material**	**Solvent**
Anode	PEDOT:PSS	shared
HIL	PEDOT:PSS	Isopropanol
HTL	TAPC	THF
EML	PVK	o-Xylene
ETL	ZnO-NPs	Isopropanol
Cathode	Ag-NPs	DMF
**Stack 4 (Score: 14.59)**		
**Layer**	**Material**	**Solvent**
Anode	PEDOT:PSS	shared
HIL	PEDOT:PSS	Water
HTL	TAPC	THF
EML	PVK	Chlorobenzene
ETL	ZnO-NPs	Methanol
Cathode	Ag-NPs	DMF
**Stack 5 (Score: 14.59)**		
**Layer**	**Material**	**Solvent**
Anode	PEDOT:PSS	shared
HIL	PEDOT:PSS	Water
HTL	TAPC	THF
EML	PVK	o-Xylene
ETL	ZnO-NPs	Ethanol
Cathode	Ag-NPs	DMF

**Table 22:** Generated stack similar to the previously analyzed one.

Layer	Material	Solvent
Anode	PEDOT:PSS	shared
HIL	PEDOT:PSS	shared
HTL	PEDOT:PSS	Isopropanol
EML	PVK	Dichlorobenzene
ETL	PO-T2T	Isopropanol
Cathode	Ag-NPs	Water

**Table 23:** Experimental OLED stack reported by Amruth et al. [31].

Layer	Material	Solvent
Anode	ITO	(Substrate)
HIL	PEDOT:PSS	shared
HTL	PEDOT:PSS	Water
EML	Super Yellow	Toluene
EIL/ETL	PFN	1-butanol
Cathode	Ag	None (VTE)

**Table 24:** Comparison between experimental stack and generated stack.

Layer	Amruth et al. [31]		Monte Carlo Output	
	Material	Solvent	Material	Solvent
Anode	ITO	-	PEDOT:PSS	shared
HIL	PEDOT:PSS	shared	PEDOT:PSS	shared
HTL	PEDOT:PSS	Water	PEDOT:PSS	Water
EML	Super Yellow	Toluene	MEH-PPV	Toluene
EIL/ETL	PFN	1-butanol	PFN	1-butanol
Cathode	Ag (Bulk)	VTE	Ag-NPs	DMF

## Data Availability

The data presented in this study are available within the article. The optimization logic and scoring parameters are fully described in Section 8: Monte Carlo Optimization.

## Abbreviations

The following abbreviations are used in this manuscript:

Ag-NPs	Silver Nanoparticles
AMOLED	Active-Matrix Organic Light-Emitting Diode
CB	Chlorobenzene
CIE	Commission Internationale de l’Éclairage
CRI	Color Rendering Index
CCT	Color Correlated Temperature
DMF	Dimethylformamide
DMSO	Dimethyl Sulfoxide
EIL	Electron Injection Layer
EML	Emissive Layer
EQE	External Quantum Efficiency
ETL	Electron Transport Layer
HIL	Hole-Injection Layer
HOMO	Highest Occupied Molecular Orbital
HSP	Hansen Solubility Parameters
HTL	Hole-Transport Layer
HUD	Head-Up Display
IPA	Isopropanol

IQE	Internal Quantum Efficiency
ITO	Indium Tin Oxide
LCD	Liquid Crystal Display
LED	Light-Emitting Diode
LUMO	Lowest Unoccupied Molecular Orbital
MEH-PPV	Poly[2-methoxy-5-(2-ethylhexyloxy)-1,4-phenylenevinylene]
Mg-Ag	Magnesium-Silver
OLED	Organic Light-Emitting Diode
PANI	Polyaniline
PEDOT:PSS	Poly(3,4-ethylenedioxythiophene) polystyrene sulfonate
PEN	Polyethylene Naphthalate
PET	Polyethylene Terephthalate
PFN	Poly [(9,9-bis(3′-(*N*,*N*-dimethylamino)propyl)-2,7-fluorene)-alt-2,7-
	(9,9–dioctylfluorene)]
PFN-Br	Poly(9,9-bis(3′-(*N*,*N*-dimethyl)-*N*-ethylammonium-propyl-2,7-fluorene)-alt-2,7-
	(9,9-dioctylfluorene))dibromide
PMOLED	Passive-Matrix Organic Light-Emitting Diode
PO-T2T	(1,3,5-Triazine-2,4,6-triyl)tris(benzene-3,1-diyl)tris(diphenylphosphine oxide)
PVK	Polyvinylcarbazole
QLED	Quantum Dot Light-Emitting Diode
TADF	Thermally Activated Delayed Fluorescence
TAPC	1,1-Bis[(di-4-tolylamino)phenyl]cyclohexane
TFT	Thin-Film Transistor
TiO_2_	Titanium Dioxide
TPBi	2,2′,2″-(1,3,5-Benzinetriyl)-tris(1-phenyl-1-H-benzimidazole)
TPD	*N*,*N*′-Bis(3-methylphenyl)-*N*,*N*′-diphenylbenzidine
VTE	Vacuum Thermal Evaporation
ZnO	Zinc Oxide
